# Ecosystem-based fisheries management: Perception on definitions, implementations, and aspirations

**DOI:** 10.1371/journal.pone.0190467

**Published:** 2018-01-30

**Authors:** John T. Trochta, Maite Pons, Merrill B. Rudd, Melissa Krigbaum, Alexander Tanz, Ray Hilborn

**Affiliations:** 1 School of Aquatic and Fishery Sciences, University of Washington, Seattle, Washington, United States of America; 2 School of Marine and Environmental Affairs, University of Washington, Seattle, Washington, United States of America; Department of Agriculture and Water Resources, AUSTRALIA

## Abstract

Ecosystem-based fisheries management (EBFM) was developed to move beyond single species management by incorporating ecosystem considerations for the sustainable utilization of marine resources. Due to the wide range of fishery characteristics, including different goals of fisheries management across regions and species, theoretical best practices for EBFM vary greatly. Here we highlight the lack of consensus in the interpretation of EBFM amongst professionals in marine science and its implementation. Fisheries policy-makers and managers, stock assessment scientists, conservationists, and ecologists had very different opinions on the degree to which certain management strategies would be considered EBFM. We then assess the variability of the implementation of EBFM, where we created a checklist of characteristics typifying EBFM and scored fisheries across different regions, species, ecosystems, and fishery size and capacity. Our assessments show fisheries are unlikely to meet all the criteria on the EBFM checklist. Consequentially, it is unnecessary for management to practice all the traits of EBFM, as some may be disparate from the ecosystem attributes or fishery goals. Instead, incorporating some ecosystem-based considerations to fisheries management that are context-specific is a more realistic and useful way for EBFM to occur in practice.

## Introduction

Fishery effects on social-ecological systems are far-reaching and complex, with the potential for mismanagement leading to severe ecosystem impacts. For example, overfishing led to the collapse of Atlantic cod (*Gadus morhua*) off Labrador and northeastern Newfoundland in 1991 [1]. While the fishing moratorium was expected to lead to Atlantic cod population recovery, population growth from a drastically reduced state was slower than expected [2] due to competition with a shellfish-dominated ecosystem [3]. Change in ecosystem structure due to heavy exploitation may leave communities more vulnerable to invasion [4]. For example, due to trophic interactions between the invasive lionfish and native reef fish predators in the Gulf of Mexico, reducing harvest of commercially valuable snapper and grouper species may lead to lower lionfish densities in the future [5].

Traditional fisheries management has focused on single species sustainability for commercially valuable species. Single-species management can be quite successful [6], but often ignores important ecosystem considerations such as species interactions, bycatch, changes in ecosystem structure, and gear impacts on habitat [7]. For example, large bycatch of Pacific halibut in the Bering Sea and Aleutian Islands Groundfish fishery have reduced the catch in the commercial Bering Sea halibut fishery [8,9]. Additionally, the use of single-species reference points, such as harvesting each species at its maximum sustainable yield (MSY), could cause severe deterioration in trophic levels and ecosystem structure by removing top predators with unpredicted consequences at bottom trophic levels [10].

To address these issues, ecosystem-based fisheries management (EBFM) has been proposed as a holistic way of managing fisheries, considering the complex dynamics between target and non-target species and the greater social-ecological system [11–14]. The U.S. National Oceanic and Atmospheric Administration [15] and the Food and Agriculture Organization (FAO) [16] define ecosystem-based management as:

“an approach that takes major ecosystem components and services (both structural and functional) into account in managing fisheries. It values habitat, embraces a multispecies perspective, and is committed to understanding ecosystem processes. Its goal is to rebuild and sustain populations, species, biological communities, and marine ecosystems at high levels of productivity and biological diversity so as not to jeopardize a wide range of goods and services from marine ecosystems while providing food, revenue, and recreation for humans” [17].

However, translating these generic statements into concise management approaches and goals, and targeted actions has inevitably led to different perspectives on EBFM definitions [18] and implementation in different regions of the world [19,20]. Thus, ongoing research objectives on EBFM have rapidly evolved from ‘what it is’ to ‘why we apply it’ to ‘how we apply it’ [18,21].

This research is just the continuing effort to reconcile the greatly varying perspectives on which scenarios constitute EBFM. The most frequently mentioned principles in the ecosystem-based management conceptual and theoretical literature include: consideration of ecosystem connections, use of scientific knowledge, stakeholder involvement, maintenance of biodiversity and acknowledgment of uncertainty [22]. Other papers advise key goals and priorities of EBFM [23], and general EBFM actions and considerations [24] to clarify EBFM implementation. Moreover, EBFM implementation performances have been quantitatively evaluated in different countries, but not in different fisheries [19,20].

The objectives of this paper are to 1) highlight the lack of consensus on what constitutes EBFM and 2) explore varying degrees of implementation of EBFM in different fisheries around the world. We hope that understanding the variations in which EBFM is perceived and applied can highlight areas for improvement.

## Methods

### Perception

To see how EBFM is conceptually perceived, we surveyed experts in different specialties of fisheries science and management on whether they consider a range of management scenarios to be EBFM. The respondents were recruited to participate in the study in person and all of them verbally consented to respond to the survey (n = 27).

**Fig 1:**
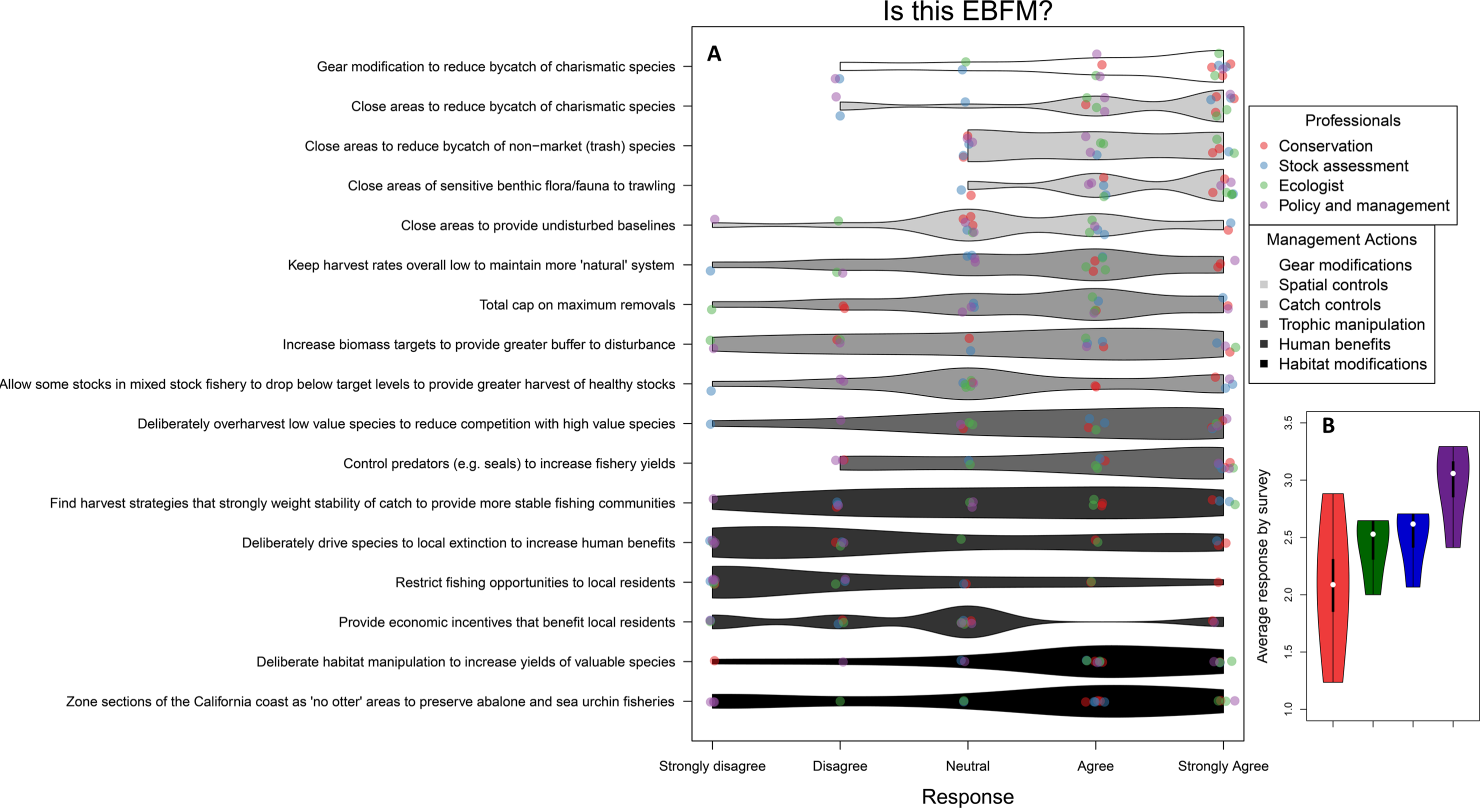
A. Survey results for defining EBFM. The y-axis is the list of scenarios asked and the x-axis the score of the final response. We divided the scenarios by categories for different management actions (gray shading) and the respondents by profession or background (colored). Each tube represents the range of responses. **B**. Shows the average responses by survey for each respondent background.

We designed the survey based on authors’ experiences and perceptions of what generates debates and confusion when talking about EBFM in different fields, especially in the U.S. The survey consisted of 17 questions categorized in different areas: gear modifications, spatial controls, catch controls, trophic manipulation, human benefits and habitat modifications (Fig 1). Answers required ordinal scores ranking each scenario from 1 to 5, from strongly agree (1) to strongly disagree (5). All responses were assigned the same weight. Respondents to our survey were highly trained U.S. professionals in stock assessment, conservation, ecology, and policy/management, whose work directly involves or is related to EBFM approaches. Based on this pool of respondents, the results reflect only variations in perception among U.S. scientists and cannot necessarily be generalized to other regions.

### Implementation

To explore EBFM implementation in different fisheries around the world, we developed a checklist of characteristics to reflect how EBFM is implemented. The checklist had 18 criteria selected from three key subjects: ecosystem, society, and management processes (Table 1). Examples of the criteria selected from the ecosystem subject area include “presence of ecosystem-based goals”, “available ecosystem models” and “fisheries independent data collection and monitoring of more than target species”. An example of criteria from the society subject area is “goals emerge from a participatory process”, and an example of management process criteria is “process for evaluation and adaptability of the management plan”. We used this checklist to calculate a unique score summarizing how many EBFM principles are part of different fisheries management plans (S1 File), not how EBFM is implemented or enforced.

**Table 1:** EBFM scoring criteria. Criteria used to score the fisheries listed in S1 File and justification for each scoring criteria.

**#**	Criteria	0	0.5	1
11	Management plan defines the bounds of the ecosystem	Bounds of ecosystem not mentioned	Bounds set poorly, not reflective of ecosystem	Full trophic and spatial considerations
12	Ecosystem-based goals	No mention of ecosystem goals	Non-specific ecosystem goals	Specific ecosystem goals
13	Goals emerge from participatory process	No participatory process	Stakeholders involved but not directly in decision-making	Stakeholders involved in decision-making
44	Considers the impact on humans (economic, cultural, social)	No social consideration	Social or economic impacts considered	Uses social-ecological-systems or other social-ecological-economic system
45	Process for evaluation and adaptability of the management plan	No built-in adaptability or evaluation	Local level legislative adaptability and evaluation	Single agency evaluation and adaptability
66	Management plan recognizes uncertainty and makes allowances	Does not acknowledge uncertainty	Takes some uncertainty into account	Provides scenarios for uncertainty and evaluates how scenarios will impact management in the future
77	Interaction of multiple species are considered	Single-species	Multiple species including non-targeted species	Ecosystem models with species/age components
88	Tradeoffs in ecosystem services are evaluated	No mention of ecosystem services	Ecosystem services are identified but not measured	Ecosystem services identified and trade-offs measured
99	Specific ecosystem targets	No mention of ecosystem targets	Ecosystem targets are identified but not evaluated	All ecosystem targets defined and evaluated
110	Fisheries-independent data collection and monitoring of more than target species	No independent data collection available	Independent data collection is available only for target species	Independent data collection available for target and non-target species
111	Harvest control rules including non-target species	No harvest control rules for non-target species	Mentions harvest controls on non-target species, but no rules stated	Separate harvest control rules for non-target species included
112	Evidence that regulations are effectively enforced	No evidence	Mentions how regulations are enforced (e.g. listed resources such as boats and workforce)	Evidence that regulations are effective (e.g. clear knowledge of illegal activity and listed enforcement actions to combat this)
113	Bycatch is monitored	No mention of bycatch observations	Bycatch is acknowledged, but not well-quantified	Bycatch rates well-defined through monitoring (e.g. full observer program)
114	Bycatch is minimized	No mention of effort to minimize or reduce bycatch	Actions to reduce bycatch (e.g. gear restrictions, area closures, timing restrictions) are considered	Enforced actions to reduce bycatch are successful
115	Sensitive habitats are identified and mapped	No mention of sensitive habitats	Potential sensitive habitats are identified but not adequately mapped	Sensitive habitats are identified and mapped
116	Sensitive habitats are protected	No mention of sensitive habitats	Sensitive habitats are protected but some use is still allowed	Sensitive habitats are protected from all use
117	Ecosystem models are available	No ecosystem models are available	Ecosystem models are available for strategic use (explore ecosystem dynamics)	Ecosystem models are available for tactical use (explore policies)
118	Ecosystem models are used in evaluating policies	No ecosystem models are available	Ecosystem models are used to strategically evaluate policies	Ecosystem models are used to tactically evaluate policies

We assessed 20 fisheries with publicly available management plans from around the world to compare EBFM implementation scores. These fisheries included fish and invertebrates, multi-species and single-species, and nationally and regionally managed. While these fisheries may not explicitly indicate EBFM as part of their management plans, we examined the extent to which EBFM principles were described in the management plans. References to these fisheries and management plans can be found in S1 File. Score values of 1, 0.5, and 0 were assigned to denote whether a criterion was met, partially met, or not met at all, respectively (Table 1). In the S1 File we also presented a description of how each score was assigned for each fishery.

## Results and discussion

### How is EBFM perceived?

The distributions of the respondents’ scores for most scenarios were broad (Fig 1A). Most of the scenarios had responses ranging from ‘strongly agree’ to ‘strongly disagree’, indicating a lack of consensus on which actions may be elements of EBFM. This was particularly true for questions regarding catch controls, trophic manipulation, human benefits, and habitat modifications (Fig 1A). These attributes may be controversial in many fisheries, such as deliberately overharvesting low value species to reduce competition with high value species to provide economic incentives that benefit local residents. Most respondents agreed that gear modifications and spatial controls fall under the umbrella of EBFM (Fig 1A). These management strategies are commonly used for high-profile ecosystem-related issues, including avoiding bycatch of charismatic species and implementing marine protected areas, and are often used in U.S. fisheries on both coasts to reduce fishing impacts on marine ecosystems [25–27]. Policy makers had very different views on the subjects than conservationists, ecologists, and stock assessment scientists, generally attributing fewer management scenarios as EBFM (higher scores) compared to other groups (Fig 1B). Conservationists showed the broadest range of responses, but in general were more willing to categorize a management scenario as EBFM (lower scores; Fig 1B).

### How is EBFM implemented in different fisheries?

**Fig 2:**
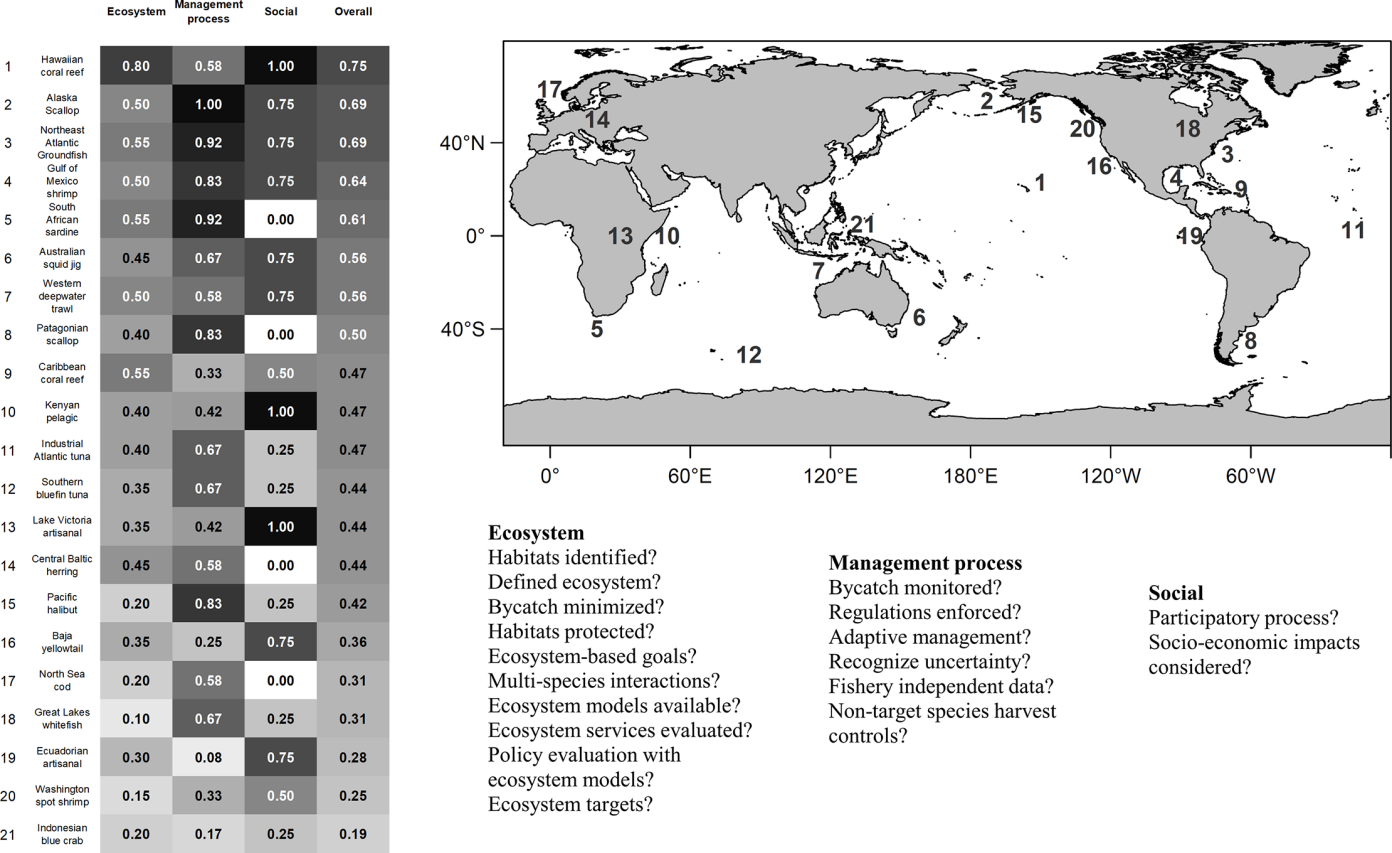
Summary table of average criteria scores. Scores belonging to either the ecosystem, social, or management process categories in columns and for each fishery in rows. The criteria belonging to these categories are listed to the bottom-right of the figure. Shading represents the relative magnitude of the score in each column (e.g. a gradient from white for 0, to black for 1). The font colors are different within the table to provide contrast with the background. The map at the top right shows the geographical locations of the fisheries considered in the study.

We did not find specific patterns in EBFM implementation scores within specific countries or regions (see map in Fig 2). The highest scoring fisheries of those reviewed included the Hawaiian Coral Reef fisheries, the Alaska Scallop fishery, and the Northeast Groundfish fishery (Fig 2 and S1 Fig). While the three top-scoring fishing were U.S. fisheries, not all U.S. fisheries scored highly, with the Washington Spot Shrimp fishery and Great Lakes Whitefish fishery scoring very poorly. However, the lowest scoring fisheries (Fig 2) were those within developing nations (e.g. Ecuadorian Artisanal fishery and Indonesian Blue Crab fishery) or with no cohesive management between multiple agencies and jurisdictions (e.g. Pacific halibut). Ecosystem considerations were at a lower priority or implemented unsuccessfully in scenarios with other barriers to successful management, including low enforcement capacity, management bodies detached from the needs of the fishing community, or joint management bodies with conflicting objectives (e.g. large-scale tuna fisheries managed by regional organizations [28])

A rigorous management plan that includes ecosystem considerations does not necessarily mean that EBFM is implemented and enforced. In 2009, Mora et al. [20] analyzed to what extent different attributes of fisheries management were related to the actual sustainability of fisheries. They found that the scientific basis on which management recommendations are made did not influence the sustainability of fisheries. They suggested that in policy makers may override scientific advice due to socioeconomic costs, political pressures, or corruption. For example, the Northeast Groundfish fishery was the third highest scoring fishery in our checklist for implementing ecosystem approaches, with the highest mark for its management process, and mid-range scores for its social and ecosystem components (Fig 2). This multispecies fishery has a very long history of management that has resulted in large increases in groundfish biomass over time. However, the Northeast Groundfish fishery management has been largely unsuccessful in enabling the recovery of cod stocks [29,30]. In this case, strong single and multispecies management with ecosystem considerations have not equated to successful management of all Groundfish components. While management regulations appeared to be well enforced in this fishery (S1 Fig), multispecies fisheries management is complex due to difficulty avoiding bycatch overlapping in time and space with target species. In general, EBFM as a goal in a management plan is not the same as a successful EBFM [31].

## Conclusions

Given the wide range of opinions, implementations, and outcomes of EBFM, managers and policy makers may want to view EBFM as a set of tools that can be used for successful management in different contexts. This perspective parallels a common legal analogy: the bundle of sticks. In the classic analogy, each stick represents a certain right that make up a larger whole, such as property rights. Through legal processes, some sticks can be removed, but the bundle still provides certain rights to an individual [32]. In the EBFM analogy, each stick represents a component of management, and an entity can create an effective management system by selecting some sticks to use without needing to use the entire bundle. Some jurisdictions and agencies may only have the capability to manage one species at a time, while others may adopt ecosystem considerations at the risk of short-term economic hardships to increase long-term sustainability. The bundle of sticks analogy provides a way to take advantage of the differences in opinions by allowing fisheries management agencies to decide which set of sticks constitutes EBFM in their context. Some of the management plans that scored very well across our EBFM metrics, such as the Northeast Groundfish fishery, have been unsuccessful at maintaining all stocks in a healthy condition. This suggests that simply utilizing more components of EBFM does not guarantee long-term successful management. Instead, it is the careful selection of which components should be applied to specific management that makes for successful fisheries.

There are multiple studies that have evaluated the implementation of EBFM at a global scale [14,20,33,34]. In general, all agree that what is intended on paper in different management plans is not always what is enforced [30]. However, our study calls attention to the point that there is no all-encompassing definition of what constitutes EBFM and this should be discussed when evaluating the EBFM performance of different fisheries. No two fisheries or ecosystems are exactly alike. Consequently, EBFM implementation is and should be context-specific, depending on local goals for management and adopting only ecosystem and societal considerations that are motivated from the understanding of the local system. In this way, management agencies selectively adopt and develop their own optimized version of EBFM from a “bundle of sticks.” The results presented here can help refocus the discussion on EBFM, instead of trying to holistically define concepts and prescribe generic approaches. This may be the best means for EBFM to achieve both sustainable fisheries and healthy marine ecosystems.

## Supporting information

S1 Fig EBFM implementation scores. EBFM implementation scores for each fishery (rows) in various categories (columns). Score values of 0, 0.5, and 1 were used to denote whether a criterion was met (1 = black), partially met (0.5 = gray), or not met at all (0 = white). The final row and column are the average fishery and criterion scores. The rows are sorted by average criterion scores from highest to lowest, and columns sorted by fishery scores from highest to lowest.
(TIF)

S1 File Criteria and references used to score each fishery. References and justifications used to score fisheries according to EBFM criteria listed in Table 1.
(PDF)

## References

[1] Myers RA, Hutchings JA, Barrowman NJ. Why do fish stocks collapse? The example of cod in Atlantic Canada. Ecological Applications. 1997 pp. 91–106.

[2] Rose GA, Rowe S. Northern cod comeback. Can J Fish Aquat Sci. 2015;72: 1789–1798. doi: 10.1139/cjfas-2015-0346

[3] Myers RA, Worm B. Rapid worldwide depletion of predatory fish communities. Nature. 2003;423: 280–283. doi: 10.1038/nature01610 12748640 10.1038/nature01610

[4] Planque B, Fromentin JM, Cury P, Drinkwater KF, Jennings S, Perry RI, et al How does fishing alter marine populations and ecosystems sensitivity to climate? J Mar Syst. Elsevier B.V.; 2010;79: 403–417. doi: 10.1016/j.jmarsys.2008.12.018

[5] Chagaris D, Binion S, Bogdanoff AK, Dahl K, Granneman J, Harris H, et al An ecosystem-based approach to evaluating impacts and management of invasive lionfish. 2017; in press.

[6] Quinn TJ, Collie JS. Sustainability in single-species population models. Philos Trans R Soc Lond B Biol Sci. The Royal Society; 2005;360: 147–62. doi: 10.1098/rstb.2004.1577 15713594 10.1098/rstb.2004.1577 PMC1636109

[7] Link JJS. What does ecosystem-based fisheries management mean? Fisheries. 2002;27: 18–21. Available: http://www.scopus.com/inward/record.url?eid=2-s2.0-1942490119&partnerID=40&md5=dc5321eb52a4acd1ddaaaa7072670c40%5Cnhttp://www.nmfs.noaa.gov/pr/sars/improvement/pdfs/meaning.pdf

[8] Witherell D, Pautzke C, Fluharty D. An ecosystem-based approach for Alaska groundfish fisheries. ICES J Mar Sci. 2000;57: 771–777. doi: 10.1006/jmsc.2000.0719

[9] Trumble RJ, Kaimmer SM, Williams GH. Estimation of Discard Mortality Rates for Pacific Halibut Bycatch in Groundfish Longline Fisheries. North Am J Fish Manag. 2000;20: 931–939. doi: 10.1577/1548-8675(2000)020<0931:EODMRF>2.0.CO;2

[10] Walters C, Christensen V, Martell S, Kitchell J. Possible ecosystem impacts of applying MSY policies from single-species assessment. ICES J Mar Sci. 2005;62: 558–568. doi: 10.1016/j.icesjms.2004.12.005

[11] Brodziak J, Link J. Ecosystem-based fisheries management: what is it and how can we do it? Bull Mar Sci. 2002;70: 589–611. doi:c:\docs\qual

[12] Janssen MA, Walker BH, Langridge J, Abel N. An adaptive agent model for analysing co-evolution of management and policies in a complex rangeland system. Ecol Modell. 2000;131: 249–268. doi: 10.1016/S0304-3800(00)00256-8

[13] Ostrom E. A general framework for analyzing sustainability of social-ecological systems. Science. American Association for the Advancement of Science; 2009;325: 419–422. doi: 10.1126/science.1172133 19628857 10.1126/science.1172133

[14] Pitcher TJ, Lam ME, Ainsworth C, Martindale A, Nakamura K, Perry RI, et al Improvements to Rapfish: A rapid evaluation technique for fisheries integrating ecological and human dimensionsa. J Fish Biol. 2013;83: 865–889. doi: 10.1111/jfb.12122 24090552 10.1111/jfb.12122

[15] Blackhart K, Stanton D, Shimada A. NOAA Fisheries Glossary. NOAA Tech Memo NMFS-F/SPO-69. 2006; 71 doi: 10.1016/j.marpolbul.2011.09.032

[16] Food and Agriculture Organization. Fishery Glossary, Terminology(A9.1FI), Conference Programming and Documentation Service. FAO Fisheries and Aquaculture Department, Rome.; 2014.

[17] U.S. National Research Council. A report of the Committee on Ecosystem Management for Sustainable Fisheries Ocean Studies Board, Commission on Geosciences, Environment and Resources, National Research Council. Washington, D.C.: National Academy Press; 1998.

[18] Link JS, Browman HI. Introduction Operationalizing and implementing ecosystem-based management. 2017;74: 379–381. doi: 10.1093/icesjms/fsw247

[19] Pitcher TJ, Kalikoski D, Short K, Varkey D, Pramod G. An evaluation of progress in implementing ecosystem-based management of fisheries in 33 countries. Mar Policy. 2009;33: 223–232. doi: 10.1016/j.marpol.2008.06.002

[20] Mora C, Myers RA, Coll M, Libralato S, Pitcher TJ, Sumaila UR, et al Management Effectiveness of the World’s Marine Fisheries. PLOS Biol. 2009;7: 1–11. doi: 10.1371/journal.pbio.1000131 19547743 10.1371/journal.pbio.1000131 PMC2690453

[21] Patrick WS, Link JS. Myths that Continue to Impede Progress in Ecosystem-Based Fisheries Management. Fisheries. 2015; 155–160. doi: 10.1080/03632415.2015.1024308

[22] Long RD, Charles A, Stephenson RL. Key principles of marine ecosystem-based management. Mar Policy. 2015;57: 53–60. doi: 10.1016/j.marpol.2015.01.013

[23] Pikitch EK, Santora C, Babcock EA, Bakun A, Bonfil R, Conover DO, et al Ecology. Ecosystem-based fishery management. Science. American Association for the Advancement of Science; 2004;305: 346–7. doi: 10.1126/science.1098222 15256658 10.1126/science.1098222

[24] Francis RC, Hixon MA, Clarke ME, Murawski SA, Ralston S. Ten Commandments for Ecosystem-Based Fisheries Scientists. Fisheries. 2007;32: 217–233. doi: 10.1577/1548-8446(2007)32[217:TCFBFS]2.0.CO;2

[25] Melvin EF, Parrish JK, Conquest LL. Novel Tools to Reduce Seabird Bycatch in Coastal Gillnet Fisheries. Conserv Biol. Blackwell Science Inc; 1999;13: 1386–1397. doi: 10.1046/j.1523-1739.1999.98426.x

[26] Broadhurst MK. Modifications to reduce bycatch in prawn trawls: A review and framework for development. Rev Fish Biol Fish. Kluwer Academic Publishers; 2000;10: 27–60. doi: 10.1023/A:1008936820089

[27] Halpern BS, Lester SE, McLeod KL. Placing marine protected areas onto the ecosystem-based management seascape. Proc Natl Acad Sci. National Acad Sciences; 2010;107: 18312–18317. doi: 10.1073/pnas.0908503107 20176945 10.1073/pnas.0908503107 PMC2972932

[28] Gilman E, Passfield K, Nakamura K. Performance of regional fisheries management organizations: ecosystem-based governance of bycatch and discards. Fish Fish. 2014;15: 327–351. doi: 10.1111/faf.12021

[29] Collie J, Minto C, Worm B, Bell R. Predation on Prerecruits Can Delay Rebuilding of Depleted Cod Stocks. Bull Mar Sci. University of Miami—Rosenstiel School of Marine and Atmospheric Science; 2013;89: 107–122.

[30] Pershing AJ, Alexander MA, Hernandez CM, Kerr LA, Le Bris A, Mills KE, et al Slow adaptation in the face of rapid warming leads to collapse of the Gulf of Maine cod fishery. Science (80-). 2015;350: 809–12. doi: 10.1126/science.aac9819 26516197 10.1126/science.aac9819

[31] Essington TE, Levin PS, Anderson L, Bundy A, Carothers C, Coleman F, et al Building effective fishery ecosystem plans—A report from the Lenfest Fishery Ecosystem Task Force Lenfest Ocean Program, Washington, D.C. [Internet]. 2016 Available: http://www.lenfestocean.org/en/news-and-publications/fact-sheet/executive-summary-building-effective-fishery-ecosystem-plans

[32] Baron JB. Rescuing the Bundle-of-Rights Metaphor in Property Law. 82 U Cin L Rev. 2014; Available: http://scholarship.law.uc.edu/uclr/vol82/iss1/2

[33] Bundy A, Chuenpagdee R, Boldt JL, de Fatima Borges M, Camara ML, Coll M, et al Strong fisheries management and governance positively impact ecosystem status. Fish Fish. 2017;18: 412–439. doi: 10.1111/faf.12184

[34] Cullis-Suzuki S, Pauly D. Failing the high seas: A global evaluation of regional fisheries management organizations. Mar Policy. 2010;34: 1036–1042. doi: 10.1016/j.marpol.2010.03.002

