# Supplementary figures and images for: Ecosystem-based fisheries management: Perception on definitions, implementations, and aspirations
Source: PLoS One. 2018 Jan 30;13(1):e0190467. doi: 10.1371/journal.pone.0190467 (PMC5790216; doi:10.1371/journal.pone.0190467)

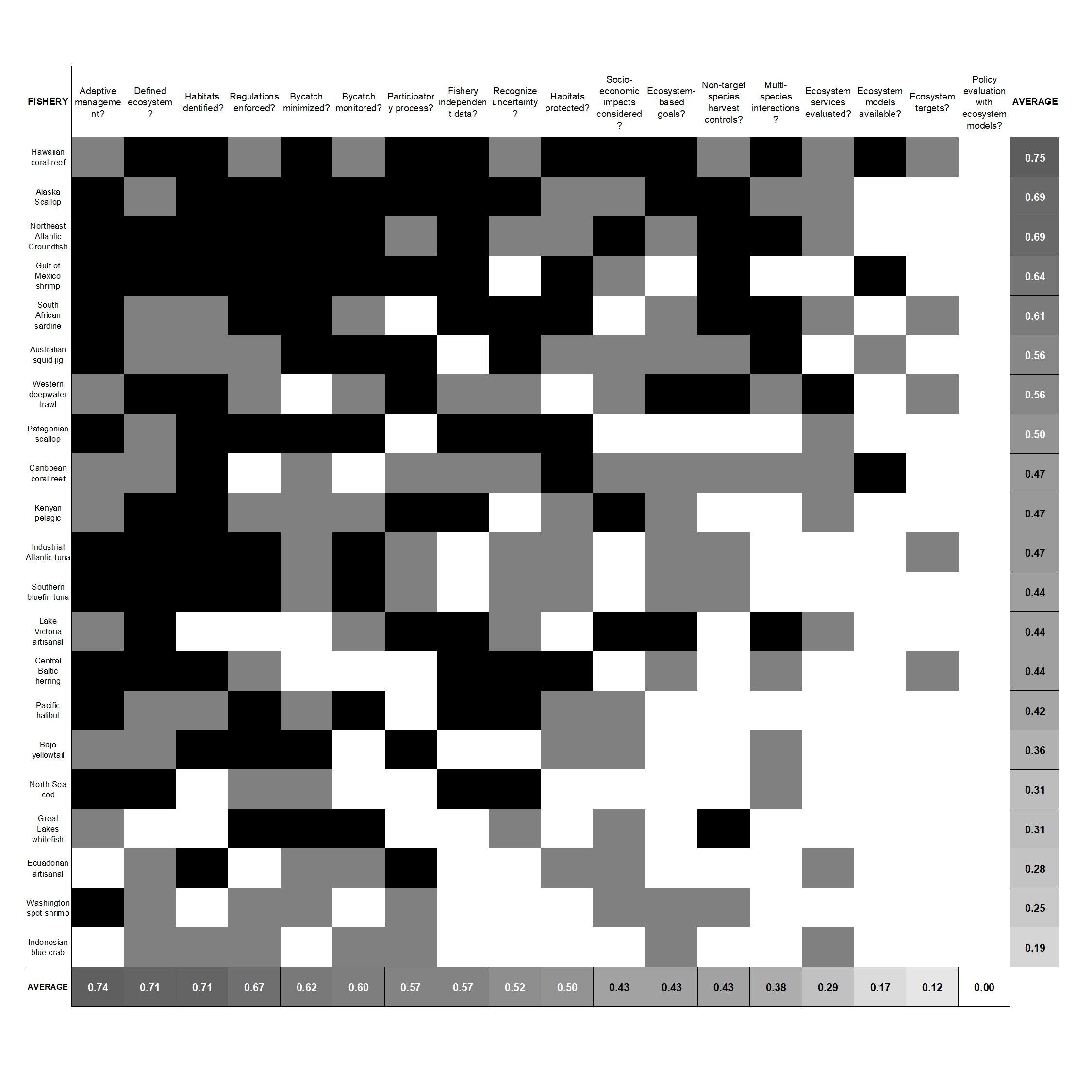

Supplement: S1 Fig — EBFM implementation scores for each fishery (rows) in various categories (columns). Score values of 0, 0.5, and 1 were used to denote whether a criterion was met (1 = black), partially met (0.5 = gray), or not met at all (0 = white). The final row and column are the average fishery and criterion scores. The rows are sorted by average criterion scores from highest to lowest, and columns sorted by fishery scores from highest to lowest. (TIF) [file pone.0190467.s001.tif]
