# Supplementary material for: Ecosystem-based fisheries management: Perception on definitions, implementations, and aspirations
Source: PLoS One. 2018 Jan 30;13(1):e0190467. doi: 10.1371/journal.pone.0190467 (PMC5790216; doi:10.1371/journal.pone.0190467)
Supplement: S1 File — References and justifications used to score fisheries according to EBFM criteria listed in Table 1. (PDF) [file pone.0190467.s002.pdf]

1. Hawaiian Coral Reef Fishery

West Pacific Fishery Management Council. 2010. Fishery Ecosystem Plan for the Hawaii Archipelago. Available at: http://www.wpcouncil.org/fishery-plans-policies-reports/hawaii-fishery-ecosystem-plan/.

West Pacific Fishery Management Council. 2016. Fishery Ecosystem Plan for the Hawaii Archipelago. Available at: http://www.wpcouncil.org/fishery-plans-policies-reports/hawaii-fishery-ecosystem-plan/.

1) Management plan defines the bounds of the ecosystem

Score: 1 – Full trophic and spatial considerations. The West Pacific Fishery Management Council switched in the mid-2000s from species/complex-based fishery management plans (FMPs) to place-based fishery ecosystem plans (FEPs). Specifically citing the goal of EBFM, the revised FEP delineates the boundaries of the Hawaiian Archipelago FEP and management unit species.

2) Ecosystem-based goals

Score: 1 – Specific ecosystem goals. Specifically citing the goal of EBFM, the revised FEP states four goals including management of target and non-target stocks, protecting species and habitats of special concern, understanding and accounting for important ecosystem parameters and their linkages, and meeting the needs of fishermen, their families, and communities in the Hawaiian archipelago.

3) Goals emerge from participatory process

Score: 1 – Stakeholders involved in decision-making. A key objective of the FEP is to increase traditional and local knowledge in decision-making by identifying relevant indigenous and local practices and knowledge that may improve scientific inquiry, utilize cultural practitioners, concepts, and bodies in the analysis of management alternatives, and utilize fishermen knowledge in the analysis of management alternatives. The Council established the Community Development Planning Committee to meet these goals.

4) Considers the impact on humans (economic, cultural, social)

Score: 1 – Uses social-ecological-systems or other social-ecological-economic system. The FEP acknowledges social, cultural, and economic dimensions are important to integrate in the place-based fishery ecosystem plan (WPFMC 2016, p. i). The first objective of the FEP is to support fishing communities, identifying various social and economic groups, ensuring conservation objectives are written to be as minimally-constraining as possible, selecting alternatives that minimize adverse economic impacts to fishing communities, and many more human-focused goals.

5) Process for evaluation and adaptability of management plan

Score: 0.5 – Local level legislative adaptability and evaluation. While the FEP is a place-based approach that considered social, cultural, and economic aspects of the fishery as well as the complex coral reef ecosystem, the management still falls under jurisdiction of the federal Magnuson-Stevens Act that requires annual catch limits at least at the complex level. This is likely not the best management approach for this fishery due to implementation issues for small-scale fisheries (WPFMC 2016, p. 3 outlines the tradeoff between top-down, centralized fisheries management and the council system).

6) Management plan recognizes uncertainty and makes allowances

Score: 0.5 – Takes some uncertainty into account. The Council process of setting annual catch limits (ACLs) lower or equal to the acceptable biological catch (ABC), which is lower than the overfishing limit (OFL) accounts for uncertaint). However, this accounts for observation error and process error, but not error in the implementation of management (e.g. enforcement).

7) Interaction of multiple species are considered

Score: 1 – Ecosystem models with species/age components. Ecosystem modeling in the Western Pacific Region uses dynamically parameterized simulation models.

8) Tradeoffs in ecosystem services are evaluated

Score: 0.5 – Ecosystem services are identified but not measured. NOAA Coral Reef Conservation Program funds research on coral reef ecosystems, but not totally clear how this is brought into the fishery ecosystem plan directly.

9) Specific ecosystem targets

Score: 0.5 – Ecosystem targets are identified but not evaluated. Target harvest control rules are set but it is not clear how they link between species.

10) Fisheries-independent data collection and monitoring of more than target species

Score: 1 – Independent data collection available for target and non-target species. The NOAA Coral Reef Conservation Program collects fishery-independent surveys of the coral reef ecosystem.

11) Harvest control rules including non-target species

Score: 0.5 – Mentions harvest controls on non-target species, but no rules states. Control rules are applied to stock complexes in many situations within this FEP. The FEP recognizes that individual species are affected differently in the complex-level control rule, and so fishing mortality must not exceed a level that would lead to its protection under the Endangered Species Act. However, the FEP does not state harvest control rules for non-target species specifically – they are just inherent in the complex-level control rules.

12) Evidence that regulations are effectively enforced

Score: 0.5 – Mentions how regulations are enforced (e.g. listed resources such as boats and workforce). While enforcement agencies exist, regulations are in place, and resources such as vessels and aircraft also exist, there are known problems with enforcement in many fisheries.

13) Bycatch is monitored

Score: 0.5 – Bycatch is acknowledged, but not well-quantified. Fishermen with commercial marine licenses are required to submit catch reports including catches and bycatch (including discards) on a per-trip basis, 5 days after trip completion. This is not the case for recreational fishermen.

14) Bycatch is minimized

Score: 1 – Actions to reduce bycatch (e.g. gear restrictions, area closures, timing restrictions) are considered. One of the main objectives of the FEP is to reduce bycatch, by promoting viable methods to reduce interactions with seabirds, marine mammals, sea turtles, corals, and other protected species. Many fish that are not often eaten commercially are also kept and eaten for subsistence.

15) Sensitive habitats are identified and mapped

Score: 1 – The NOAA Coral Reef Conservation Program funding identifies and refines essential fish habitat for important commercial and recreational species.

16) Sensitive habitats are protected

Score: 1 – Sensitive habitats are protected from all use. The Papahanaumokuakea National Monument protects the exclusive economic zone of the Northwestern Hawaiian Islands.

17) Ecosystem models are available

Score: 1 – Ecosystem models are available for tactical use (explore policies). There is an Ecopath model available for the Northwestern Hawaiian Islands.

18) Ecosystem models are used in evaluating policies

Score: 0 – No ecosystem models are used to evaluate policies for the Hawaiian coral reef fisheries.

2. Alaska Scallop Fishery

Norden, W. 2012. Monterey Bay Aquarium Seafood Watch. Weathervane Scallop, Patinopecten caurinus, Alaska: Gulf of Alaska, Bering Sea and Aleutian Islands. Available at: http://www.seafoodwatch.org/-/m/sfw/pdf/reports/mba_seafoodwatch_weathervanescallopreport.pdf.

NPFMC. 2014. Fishery Management Plan for the Scallop Fishery off Alaska. Anchorage. Available at: http://www.npfmc.org/wp-content/PDFdocuments/fmp/Scallop/ScallopFMP2014.pdf.

NPFMC. 2015. Stock assessment and fishery evaluation report for the weathervane scallop fishery off Alaska. Available at: http://www.npfmc.org/wp-content/PDFdocuments/membership/PlanTeam/Scallop/ScallopSAFE215.pdf.

1) Management plan defines the bounds of the ecosystem

Score: 0.5 – Bounds are set spatially by the EEZ, but there are not trophic considerations.

2) Ecosystem-based goals

Score: 1 – Plan has specific habitat objectives, and since scallops are benthic, this has the effect of ecosystem goals as well. Ecosystem considerations are included in the fisheries management plan (FMP).

3) Goals emerge from participatory process

Score: 1 – Plan arises from regional fishery council state input and stakeholders.

4) Considers the impact on humans (economic, cultural, social)

Score: 1 – Specific economic and social objectives to maximize economic and social benefits are a goal of the plan. Analysis takes into account present and future benefits, and economic impacts of changes to coastal communities.

5) Process for evaluation and adaptability of the management plan

Score: 1 – FMP is amended periodically and catch is authorized on an annual basis.

6) Management plan recognizes uncertainty and makes allowances

Score: 1 – Allowable catch is reduced due to uncertain biomass, and questionable data from previous years. Dredge surveys that show lower than optimal biomass can be closed to fishing.

7) Interaction of multiple species are considered

Score: 0.5 – Interactions are not fully understand in terms of the ecosystem, but sensitive areas are closed to dredging to reduce impacts on groundfish and crabs.

8) Tradeoffs in ecosystem services are evaluated

Score: 0.5 – Only damage of benthic habitat by fishing gear is acknowledged.

9) Specific ecosystem targets

Score: 0 – There are no specific ecosystems targets in the management plan.

10) Fisheries-independent data collection and monitoring of more than target species

Score: 1 – Observers are present on all boats outside of Cook inlet area, and Alaska Dept of Fish and Game performs annual dredge surveys to determine biomass in specific areas.

11) Harvest control rules including non-target species

Score: 1 – Annual catch limits, and bycatch limits which include potential area closure for certain types of bycatch, particularly Tanner crab.

12) Evidence that regulations are effectively enforced

Score: 1 – At sea and in port enforcement by several state and federal agencies, and stock increase after previous overfished status indicate good enforcement.

13) Bycatch is monitored

Score: 1 – At sea observers are on the vast majority of boats.

14) Bycatch is minimized

Score: 1 – Gear is designed to minimize bycatch. Halibut and crab bycatch are monitored and reported, and can result in early closure of areas to fishing.

15) Sensitive habitats are identified and mapped

Score: 1 – Large areas are designated as essential habitat.

16) Sensitive habitats are protected

Score: 0.5 – Areas are protected, but the nature of bottom dredging limits the effectiveness of protections to areas that are designated as non-fishing.

17) Ecosystem models are available

Score: 0 – No ecosystem model exists.

18) Ecosystem models are used in evaluating policies

Score: 0 – No ecosystem model exists.

3. Northeast Atlantic Groundfish Fishery

New England Fishery Management Council. 2017. Framework Adjustment 56 to the Northeast Multispecies Fishery Management Plan. Available at: http://s3.amazonaws.com/nefmc.org/170629_Groundfish_FW56_EA_resubmit.pdf

1) Management plan defines the bounds of the ecosystem

Score: 1 – The ecosystem is defined as the Northeast U.S. Shelf Ecosystem (area from the Gulf of Maine south to Cape Hatteras, extending from the coast seaward to the edge of the continental shelf, including the slope sea offshore to the Gulf Stream). Also, components of the ecosystem include the physical environment, essential fish habitat, target species, non-target species/bycatch, protected resources, and human communities.

2) Ecosystem-based goals

Score: 0.5 – No explicit EBFM goals are defined. However, some ecosystems components are considered as part of the objectives of the management plan: Goal #2 is to "create a management system so that fleet capacity will be commensurate with resource status so as to achieve goals of economic efficiency and biological conservation and that encourages diversity within the fishery". Goal #4: "minimize, to the extent practicable, adverse impacts on fishing communities and shoreside infrastructure". Goal #9: "Adopt measures consistent with the habitat provisions of the M-S Act, including identification of EFH and minimizing impacts on habitat to the extent practicable". Goal #10: "Identify and minimize bycatch, which include regulatory discards, to the extent practicable, and to the extent bycatch cannot be avoided, minimize the mortality of such bycatch".

3) Goals emerge from participatory process

Score: 0.5 – No direct references about other actors working in the definition of the management goals. However, the Council review process involves public meetings at which affected stakeholders have opportunity to provide comments on the technical reports.

4) Considers the impact on humans (economic, cultural, social)?

Score: 1 – Socio-economic factors are analyzed and considered. The MSA stipulates that the social and economic impacts to all fishery stakeholders should be analyzed for each proposed fishery management measure to provide advice to the Council when making regulatory decisions.

5) Process for evaluation and adaptability of the management plan?

Score: 1 – Regular assessments are performed and management measures are adapted based in the new information available. As a consequence, fishing communities also adapt to changes in fishery regulations.

6) Management plan recognizes uncertainty and makes allowances.

Score: 0.5 –They used a current management uncertainty buffer for non-allocated stocks of 7% in the quotas. However, other sources of uncertainty are not considered.

7) Interaction of multiple species are considered

Score: 1 – The management plan specifies the management measures for thirteen groundfish species (cod, haddock, yellowtail flounder, pollock, plaice, witch flounder, white hake, windowpane flounder, Atlantic halibut, winter flounder, redfish, Atlantic wolffish, and ocean pout) off the New England and Mid-Atlantic coasts.

8) Tradeoffs in ecosystem services are evaluated

Score: 0.5 – Some tradeoffs are considered (i.e. environment, target species, non-target species/bycatch and human communities), but not all ecosystems services have been evaluated.

9) Specific ecosystem targets

Score: 0 – There are no specific ecosystem targets in the management plan.

10) Fisheries-independent data collection and monitoring of more than target species

Score: 1 – Fisheries-independent surveys are performed each year and target and non-target species are monitored.

11) Harvest control rules including non-target species.

Score: 1 – Harvest control rules exist for all groundfish species.

12) Evidence that regulations are effectively enforced.

Score: 1 – Current catch is lower than the TACs.

13) Bycatch is monitored

Score: 1 –The primary tools used to monitor and report bycatch in this multispecies fishery are the Vessel Trip Report system (VTR) and the NEFSC Observer Program (NEFOP).

14) Bycatch is minimized

Score: 1 – Different bycatch reduction programs exist to reduce bycatch in the fisheries affecting these species (Atlantic Large Whale Take Reduction Plan, Harbor Porpoise Take Reduction Plan, and the

Bottlenose Dolphin Take Reduction Plan). There is evidence of reduction of loggerhead bycatch in bottom otter trawls. Current management measures, including those implemented through Amendment 16, are expected to continue to control effort, and decrease bycatch and discards.

15) Sensitive habitats are identified and mapped

Score: 1 – Different bycatch avoidance areas are identified and mapped (SMAST Bycatch Avoidance Program). Areas of high marine mammal bycatch and small cetacean and pinniped interactions with Northeast or Mid-Atlantic bottom trawl and gillnet gears have been identified.

16) Sensitive habitats are protected

Score: 0.5 – SMAST Bycatch Avoidance Program the system was implemented in 2010, and has continued each year with additional participating vessels and areas of bycatch reporting and avoidance.

17) Ecosystem models are available

Score: 0 – No ecosystem models are available.

18) Ecosystem models are used in evaluating policies

Score: 0 – A multispecies approach is considered, but no ecosystem models are used.

4. Gulf of Mexico shrimp Fishery

Managing fishery resources in the U.S. federal waters on the Gulf of Mexico. Shrimp management plan.
http://archive.gulfcouncil.org/fishery_management_plans/shrimp_management.php
http://gulfcouncil.org/fishery-management/implemented-plans/shrimp/

Karnauskas, M., M.J. Schirripa, C.R. Kelbe, G.S. Cook, and J.K. Craig. 2013. Ecosystem Status Report for the Gulf of Mexico. U.S. Department of Commerce. NOAA National Marine Fisheries Service. Southeast Fisheries Science Center. Available at:
http://gulfcouncil.org/docs/Gulf%20of%20Mexico%20Ecosystem%20Status%20Report.pdf

Geers, M., Pikitch E.K. and M.G. Frisk. 2016. An original model of the northern Gulf of Mexico using Ecopath with Ecosim and its implications for the effects of fishing on ecosystem structure and maturity. Deep Sea Research Part II: Topical Studies in Oceanography. 129, 319-331.

1) Management plan defines the bounds of the ecosystem

Score: 1 – Physical ecosystem bounds are defined by federal waters. Also, coasts and estuaries are mentioned to be important areas for shrimp growth. Various ecosystem components are defined in the Ecosystem status report. In this fishery different species are caught: white, brown, pink, and royal red shrimp.

2) Ecosystem-based goals

Score: 0 – Although good information exists about the Gulf of Mexico ecosystem, no specific ecosystem goals are defined for this fishery in the management plan.

3) Goals emerge from participatory process?

Score: 1 – The advisory committee includes scientific agencies, environmental NGOs, industry members, and recreational sectors.

4) Considers the impact on humans (economic, cultural, social)?

Score: 0.5 – Some socioeconomic indicators exist for this fishery. However, no cultural impact is considered. See section 8 of the Ecosystem Status Report for the Gulf of Mexico. This section offers economic values for some ecosystem components.

5) Process for evaluation and adaptability of the management plan?

Score: 1 – Annual catch limits and accountability measures exist for these fisheries. They are evaluated annually. Seasonal closures, size and trip limits are also included and adapted if necessary.

6) Management plan recognizes uncertainty and makes allowances.

Score: 0 – There is no mention of it in the management plan.

7) Interaction of multiple species are considered

Score: 0 – In this fishery multiple species are considered during the management process, white, brown, pink, and royal red shrimp. However, the interactions among them or with other species are not used for management advice.

8) Tradeoffs in ecosystem services are evaluated

Score: 0 – Ecosystem services are not evaluated in these shrimp fisheries.

9) Specific ecosystem targets

Score: 0 – Ecosystem targets are not specified in the management plan.

10) Fisheries-independent data collection and monitoring of more than target species

Score: 1 – The Gulf of Mexico Fishery Management Council monitors abundance of target and non-target species including red snappers and marine turtles. They have annually Shrimp/Groundfish Surveys.

11) Harvest control rules including non-target species.

Score: 1 – They have control rules for target species and by-catch limits for non-target species.

12) Evidence that regulations are effectively enforced.

Score: 1 – TACs are effectively enforced for target species.

13) Bycatch is monitored

Score: 1 – Bycatch is monitored through an on-board observer program.

14) Bycatch is minimized

Score: 1 – Amendment 9 addresses the issue of reducing the bycatch of juvenile red snapper in the shrimp trawl fishery. Amendment 10 requires the installation of NMFS-certified BRDs that reduce the bycatch of finfish by at least 30%.

15) Sensitive habitats are identified and mapped

Score: 1 – Yes. Marine protected areas (MPAs) in the Gulf of Mexico protect over 280,800 square kilometers of the Gulf waters. These MPAs are managed by the National Marine Fisheries Service with the Gulf of Mexico Fishery Management Council.

16) Sensitive habitats are protected

Score: 1 – Yes. See previous paragraph.

17) Ecosystem models are available

Score: 1 – An Ecopath model exist (Geers et al. 2016) in addition to an Ecosystem status report (see references).

1) Ecosystem models are used in evaluating policies

Score: 0 – Not yet fully considered.

5. South African Sardine Fishery

Peacock, S. 2011. Fishery Assessment Report. IFFO Global Standard for Responsible Supply of Fishmeal and Fish Oil. South Africa Anchovy. Available at: http://www.iffo.net/files/iffoweb/approved-raw-materials/whole-fish/anchovy-south-africa.pdf.

Coetzee, J. and A. Badenhorst. 2013. Status and Management of the South African Small Pelagic Fishery 2012. Oceana Group Limited Scientific reports. Available at: http://oceana.co.za/pdf/Status-of-the-Small-Pelagic-2016.pdf.

1) Management plan defines the bounds of the ecosystem

Score: 0.5 – The physical boundaries are defined as the South Africa coastline extending from the Orange River in the west on the border with Namibia, to Ponta do Ouro in the east on the Mozambique border. However, the trophic boundaries are not clear. Only references to the sardine - anchovy complex. Also, there are some controversies about the limits between the south and west stocks.

2) Ecosystem-based goals

Score: 0.5 – No explicit ecosystem goals are defined but they mentioned: “protect the ecosystem as a whole, including species which are not targeted for exploitation”.

3) Goals emerge from participatory process

Score: 0 – No references about other actors working in the definition of the management goals.

4) Considers the impact on humans (economic, cultural, social)

Score: 0 – Not for management purposes. There is lack of information on eco-impacts harvesting forage fish. Only some evaluations exist of the economic impacts of closing some areas but it is not considered for management purposes.

5) Process for evaluation and adaptability of the management plan

Score: 1 – Anchovy quotas are set according to the small pelagic Operational Management Procedure (OMP). The OMP is created and revised by scientific working groups. This ensures that the level of fishing is always set and adjusted according to scientific advice.

6) Management plan recognizes uncertainty and makes allowances.

Score: 1 – Yes, it is included in the OMP (e.g. mixing between stocks, stock definitions, etc).

7) Interaction of multiple species are considered

Score: 1 – The OMP achieves desired tradeoff in unintended depletion of multiple resources and mutually conflicting objectives, such as maximizing catch, minimizing catch variability, and minimizing risk of resource depletion. Also, top predators such us penguins are considered in the model. Ongoing research: use of logger technology to assess areas in which prey is caught and where birds spend majority of time at sea - model different foraging efficiency, food abundance and availability scenarios.

8) Tradeoffs in ecosystem services are evaluated

Score: 0.5 – Some tradeoffs are considered (see previous paragraph), but not all ecosystems services have been evaluated.

9) Specific ecosystem targets

Score: 0.5 – Specific ecosystem targets are not clearly specified in the management plan. However, protection of the ecosystem as a whole, including species which are not targeted for exploitation, is one of those targets.

10) Fisheries-independent data collection and monitoring of more than target species

Score: 1 – The South African fishing industry is managed and regulated by the Fisheries Management Branch (FMB) of the Department of Agriculture, Forestry and Fisheries (DAFF). The FMB conduct biannual research surveys. The hydro-acoustic survey program to estimate pelagic fish biomass was initiated in 1983.

11) Harvest control rules including non-target species.

Score: 1 – HCR exist for the target species. Also, sardine, redeye round herring and horse mackerel, are subject to annual bycatch quotas.

12) Evidence that regulations are effectively enforced.

Score: 1 – The evidence of compliance to the set TAC to this scientific advice were positive and in compliance to the precautionary approach adopted within these pelagic fisheries.

13) Bycatch is monitored

Score: 0.5. Observers on boats have been deployed in the pelagic fishery since 1999. The most significant gap in the observer data pertains to the low coverage of the pelagic fleet, which is currently around 8% of fishing trips. All landings are monitored by a government official. Samples are taken of landings every 30 minutes to check bycatch composition. Excessive bycatch of certain species leads to area closures, as described in the assessment report. However, this is only monitored at landings and not onboard.

14) Bycatch is minimized

Score: 1 – The major bycatch species in the small pelagic fishery are subject to quotas, and in any case the fishery is considered highly targeted (within the four main species caught). Small pelagic fishing permits set out a detailed plan for regional fishery closures if bycatch exceeds defined minimums, and processor permits prohibit certain gear types to minimize Cape fur seal casualties.

15) Sensitive habitats are identified and mapped

Score: 0.5 – Some areas are identified and mapped such as penguin colonies. However, no other sensitive areas are mentioned.

16) Sensitive habitats are protected

Score: 1 – The sensitive habitats identified are protected. MPAs around penguin breeding colony exist. Also, excessive bycatch of certain species leads to area closures. Vessel monitoring systems are presently on board every pelagic vessel and provide data on location (with a temporal resolution of six hours), and are used for compliance purposes to ensure that vessels do not fish in restricted areas.

17) Ecosystem models are available

Score: 0 – No ecosystem models are available.

18) Ecosystem models are used in evaluating policies

Score: 0 –A multispecies approach is considered, but no ecosystem models are used.

6. Australian Southern Squid Jig Fishery

Southern Squid Jig Fishery. Bycatch action plan. The Australian Fisheries Management Authority. 2004. Available at: www.afma.gov.au/wp-content/uploads/2010/06/squid_bap.pdf

Ecological Risk Management. Report for the Southern Squid Jig Fishery. April 2009. Available at: http://www.afma.gov.au/sustainability-environment/ecological-risk-management-strategies/ssjf_erm_apr09/

1) Management plan defines the bounds of the ecosystem

Score: 0.5 – The fishery is defined by a large area that overlaps with other fishery management schemes. The heavily fished areas highly depend on spatial abundance of squid that is not well understood, observed or predicted.

2) Ecosystem-based goals

Score: 0.5 – The FMP focuses on 'Ecologically Sustainable Development' goals of limiting bycatch, limited harm to protected species, habitats or communities. However, there are no clear baselines or quantitative goals outlined.

3) Goals emerge from participatory process

Score: 1 – SSJF Resource Assessment Group provides advice to the South East Management Advisory Committee and the AFMA Commission. Advisory committee includes scientific agencies, environmental NGOs, industry members, recreational and charter sector representatives, state governments.

4) Considers the impact on humans (economic, cultural, social)

Score: 0.5 – The Fisheries Management Act of 1991 of the AFMA includes stated objectives to 1) maximize economic efficiency, 2) be accountable to fishing industry and the Australian community. However, these objectives are not clearly operationalized in the management plan.

5) Process for evaluation and adaptability of the management plan

Score: 1 – Suites of intermediate and limit catch and effort triggers are defined based on recent catch history and determined by an assessment. When these triggers hit, they hold a special meeting and undertake full spatial and non-spatial depletion analysis.

6) Management plan recognizes uncertainty and makes allowances.

Score: 1 – The plan takes a precautionary approach, and chooses a total allowable effort well below what is determined as the maximum sustainable effort. They also use trigger limits that help avoid overfishing when squid populations are believed to be low.

7) Interaction of multiple species are considered

Score: 1 – The Ecological Risk Assessment identifies byproduct, bycatch and target species, however since the jig is highly selective, all impacts are negligible or minor. They have assessed the 216 protected species as listed as potentially impacted by the fishery and found each to be in the negligible or minor impact category.

8) Tradeoffs in ecosystem services are evaluated

Score: 0 – The Ecological Risk Assessment does not mention ecosystem services, or specify beyond catch and abundance for the fishery.

9) Specific ecosystem targets

Score: 0 – The emphasis lies on single-species management of the squid. The management documents do not specifically mention any ecosystem targets.

10) Fisheries-independent data collection and monitoring of more than target species

Score: 0 – No biological reference points or fishery independent stock assessment data was included in the management documents. Information comes from CPUE and past harvest data.

11) Harvest control rules including non-target species.

Score: 0.5 – Sets limits on non-target species in the bycatch work-plan, but they are not particularly relevant due to the highly selective nature of the gear-type.

12) Evidence that regulations are effectively enforced.

Score: 0.5 – The fishery has an observer program, but not required on-board unless the AFMA requests it. There are significant consequences for being caught violating the rules or misreporting incidental catch which may deter illegal activity.

13) Bycatch is monitored

Score: 1 – This fishery has a bycatch workplan, with the AFMA reporting on the status every 6 months, and reviewing the plan entirely every two years. Fishers are required to record catch and discards of any non-target species or interaction with protected species. Data is published on website allowing fishers to learn about common locations for bycatch issues.

14) Bycatch is minimized

Score: 1 – The documentation states that the 100kg limit of non-target species per trip is rarely reached. The gear is highly selective. The only possible concern listed is with lights confusing seabirds.

15) Sensitive habitats are identified and mapped

Score: 0.5 – The management preforms spatial depletion analysis based on catch distribution to help identify local depletion events, however, the patchy distribution of squid and fishing activity has precluded rigorous mapping of sensitive habitat.

16) Sensitive habitats are protected

Score: 0.5 – The spatial depletion analysis will identify areas with high effort and low CPUE to impose seasonal spatial closures to redirect fishery towards higher density areas. However, they do not explicitly protect spawning grounds or other sensitive habitat on a permanent or annual basis for the purpose of this fishery.

17) Ecosystem models are available

Score: 0.5 – The management documents reference that the Total Allowable Effort (TAE) is set utilizing ecosystem models, however it is not clear how developed they are, and details are lacking.

18) Ecosystem models are used in evaluating policies

Score: 0 – It is unclear from the management documents the extent to which these models are utilized, if at all, in evaluating policies.

7. Western Deepwater Trawl Fishery

Harvest Strategy for the Western deepwater trawl fishery and North West slope trawl fishery. 2011. Available at: www.afma.gov.au/wp-content/.../2011-WDTF-and-NWSTF-Harvest-Strategy.pdf

Fletcher W.J., Shaw J., Gaughan D.J. and S.J. Metcalf. 2011. Ecosystem based fisheries management case study report. West Coast Bioregion Available at: www.fish.wa.gov.au/Documents/research_reports/frr225.pdf

1) Management plan defines the bounds of the ecosystem

Score: 1 – This fishery has 4 clearly defined sub-boundaries, differentiated by effort, and based on the slopes of these deepwater areas.

2) Ecosystem-based goals

Score: 1 – The Ecological Risk Assessment and harvest plan explicitly list the objectives of:
"Avoid negative impacts on the quality of the environment."
"Avoid negative impacts on composition of the community."
"Avoid reduction in the quality of habitat".

3) Goals emerge from participatory process?

Score: 1 – The management process for this fishery begins with a scoping exercise that relies solely on stakeholder input. The selection of objectives and activities are also presented to stakeholders for modifications. The stakeholders include industry members, management, scientists, and conservationists.

4) Considers the impact on humans (economic, cultural, social)

Score: 0.5- The Fisheries Management Act of 1991 of the AFMA includes stated objectives to 1) maximize economic efficiency, 2) be accountable to fishing industry and the Australian community. However, these objectives are not clearly operationalized in the management plan.

5) Process for evaluation and adaptability of the management plan?

Score: 0.5 – Evaluation of the management plan happens every 4 years, with certain criteria assessed every year. However, this does not score a 1, as the management doesn't seem to have much ability to adapt to high levels of effort.

6) Management plan recognizes uncertainty and makes allowances.

Score: 0.5 – The management undergoes a detailed SICA evaluation of possible disturbances, with multi-levels of analysis depending on the perceived risk. However, it is unclear if these result in specific allowances.

7) Interaction of multiple species are considered

Score: 0.5 – The management undergoes the Ecological Risk Assessment that attempts to understand interaction between various species and identify what is known, however this assessment has limited data, and so has little impact on management.

8) Tradeoffs in ecosystem services are evaluated

Score: 1 – The Ecological Risk Assessment details natural processes and resources affected by the fishery. This document contains a large table of all possible habitats that could be affected beyond catch levels, since trawling has high impact.

9) Specific ecosystem targets

Score: 0.5 – The Ecological Risk Assessment does do analysis on the ecosystem level, however the targets are very general objectives rather than specific targets.

10) Fisheries-independent data collection and monitoring of more than target species

Score: 0.5 – The Ruby Snapper has a stock assessment, and additional research through academic research has aided in the monitoring, but many species lack data.

11) Harvest control rules including non-target species.

Score: 1 – The management documents include a detailed report on how to deal with various harvest control triggers depending on the species, level of catch, including non-target species, protected species and bycatch.

12) Evidence that regulations are effectively enforced.

Score: 0.5 – The observer program exists but is not 100% mandatory coverage. Reporting of incidents and catch levels are based primarily on honor system. However the documents report that the regulations are well enforced.

13) Bycatch is reported and monitored.

Score: 0.5 – Log books are fully utilized, however, without mandatory observer coverage, the fishery has a high estimated discard rate and many of the discards go unidentified.

14) Bycatch is minimized

Score: 0 – An estimated 30-45% of the catch is discarded and is a non-target species, often unidentified.

15) Sensitive habitats are identified and mapped

Score: 1 – The management documents include 48 different habitats, each assessed using habitat productivity-susceptibility analysis. These areas are then categorized as high, medium or low risk based on their geomorphology, substratum, and dominant fauna.

16) Sensitive habitats are protected

Score: 0 – They have no closed the sensitive areas based on the findings of the above analysis.

17) Ecosystem models are available

Score: 0 – There is no mention of ecosystem models. Management documents reference knowing little about the species composition of this deep ocean due to many species being unidentified.

18) Ecosystem models are used in evaluating policies

Score: 0 – There is no mention of ecosystem models. Management documents reference knowing little about the species composition of this deep ocean due to many species being unidentified.

8. **Patagonian Scallop trawl fishery**

Pottinger, R.P., J. Curelovich, E. Morsan, H.J. Cranfield, and J. Mendo. 2011. MSC assessment report: Patagonian scallop fishery. Assessed against the Principles and Criteria of the MSC. Organizacion Internacional Agropecuaria. Argentina. Available at: file:///C:/Users/Maite%20Pons/Desktop/PATAGONIAN%20SCALLOP_Public%20Certification%20Report%20-%20280817.pdf.

Management plan in Spanish available at: https://www.ecolex.org/details/legislation/resolucion-no-405-establece-un-plan-de-manejo-de-la-especie-vieira-patagonica-lex-faoc053528/

1) Management plan defines the bounds of the ecosystem

Score: 0.5 – The physical limits are defined by the EEZ of Argentina. However, no trophic or other ecosystem bounds are mentioned in the management plan.

2) Ecosystem-based goals

Score: 0 – No specific ecosystem goals are defined for this fishery in the management plan.

3) Goals emerge from participatory process

Score: 0 – There is no participatory process involved in the definition of management goals.

4) Considers the impact on humans (economic, cultural, social)

Score: 0 – No references of these impacts as part of the managements goals.

5) Process for evaluation and adaptability of the management plan

Score: 1 – It is certified by the Marine Stewardship Council (MSC). MSC monitors this fishery every year and re-assesses it every 5 years.

6) Management plan recognizes uncertainty and makes allowances.

Score: 1 – Some uncertainties are recognized and the MSC evaluates them and make allowances every year. Moreover, TACs, minimum size regulations and area closures are evaluated each year by INIDEP through independent fishery surveys.

7) Interaction of multiple species are considered

Score: 0 – There is no mention of it in the management plan.

8) Tradeoffs in ecosystem services are evaluated

Score: 0.5 – A rotational fishing strategy is implemented, so not only scallops are protected, but also other benthic invertebrate associations. However, no other services are evaluated.

9) Specific ecosystem targets

Score: 0 – Ecosystem targets are not specified in the management plan.

10) Fisheries-independent data collection and monitoring of more than target species

Score: 1 – Size range of individual by-catch species are recorded in research dredge catches during research surveys.

11) Harvest control rules including non-target species.

Score: 0 – Harvest control rules do not exist for this fishery.

12) Evidence that regulations are effectively enforced.

Score: 1 – It is monitored by MSC and recognized for being effectively enforced.

13) Bycatch is monitored

Score: 1 – On board observer programs from commercial trawl fisheries exist in Argentina. 82 non-target species captured by the fishery have been identified and categorized by feeding niche. Echinoderms, including predatory asteroids, and the herbivorous echinoids and detritivore ophiuroids, were the most important groups.

14) Bycatch is minimized

Score: 1. Bycatch is minimized by gear restrictions and area closures.

15) Sensitive habitats are identified and mapped

Score: 1 – They are identified each year by fishery surveys.

16) Sensitive habitats are protected

Score: 1 – Area closures are implemented each year in order to protect these sensitive habitats.

17) Ecosystem models are available

Score: 0 – No ecosystem models exist for this area.

18) Ecosystem models are used in evaluating policies

Score: 0 – No models exist.

9. Caribbean Coral Reef

NOAA Fisheries Southeast Regional Office. 2011. Caribbean Reef Fish Rulemakings, NOAA Sustain. Fish. Available at: http://sero.nmfs.noaa.gov/sustainable_fisheries/policy_branch/rules/caribbean/reef_fish/temp_index.html.

1) Management plan defines the bounds of the ecosystem

Score: 0.5 – Bounds set poorly, not reflective of ecosystem. The Caribbean FMP does not define ecosystem bounds, but acknowledges that the stocks (many if not most) range across state and

international boundaries). One of the FEP goals is to promote compatible or uniform management of the pan-Caribbean species.

2) Ecosystem-based goals

Score: 0.5 – Nonspecific ecosystem goals. The Caribbean is still using a fishery management plan (FMP), rather than a fishery ecosystem plan (FEP). The FMP objectives are very species-specific, although they have objectives for target and non-target species.

3) Goals emerge from participatory process

Score: 0.5 – Stakeholders involved but not directly in decision-making. The "Council has encouraged joint participation by other Caribbean nations in the preparation of FMPs", which covers international management, but not stakeholders in the fishery. Stakeholders were included in the public consultation process by public hearings and public comment (Appendix IV). Stakeholder participation is meant to be included in the Council process, but it is not specifically addressed in the FMP.

4) Considers the impact on humans (economic, cultural, social)

Score: 0.5 – The FMP describes the social and cultural framework of fishermen and their communities. The optimum yield accounts for relevant economic, social, or ecological factors (p. vii). However, the ways that these social and economic concerns are considered are not directly quantified, specified, or clearly laid out in the FMP.

5) Process for evaluation and adaptability of the management plan

Score: 0.5 – Local level legislative adaptability and evaluation. Management falls under jurisdiction of the federal Magnusson-Stevens Act that requires annual catch limits at least at the complex level. This is likely not the best management approach for this fishery due to implementation issues for small-scale fisheries.

6) Management plan recognizes uncertainty and makes allowances

Score: 0.5 – Takes some uncertainty into account. The FMP acknowledges uncertainty in calculations of MSY, and claims to use preventative management to take protective measures before a fishery is in dire trouble. However, there are many more aspects of uncertainty, as these fisheries are mostly all data-poor. The ways of setting catch limits are more thoroughly documented in later amendments, but do not adequately account for implementation error in managing these types of stocks using catch limits.

7) Interaction of multiple species are considered

Score: 0.5 – multiple species including non-targeted species. It is acknowledged that the interactions of the numerous species are poorly understood and are acknowledged in the problems estimating MSY. However, it does not appear that a solution to this problem is suggested, just that the interactions are known to exist.

8) Tradeoffs in ecosystem services are evaluated

Score 0.5 – Ecosystem services are identified but not measured. NOAA Coral Reef Conservation Program funds research on coral reef ecosystems, but this is not mentioned in the FMP.

9) Specific ecosystem targets

Score: 0 – No mention of ecosystem targets. There is no mention of ecosystem targets in the FMP.

10) Fisheries-independent data collection and monitoring of more than target species

Score: 0.5 – Independent data collection is available only for target species. Fisheries-independent data workshop and increased funding for data collection of (specifically) commercially important species.

11) Harvest control rules including non-target species

Score: 0.5 – mentions harvest controls in non-target species, but no rules stated. ACLs and accountability measures for commercially important taxa only (Final Rule to Amendment 6 of FMP).

12) Evidence that regulations are effectively enforced

Score: 0 – No evidence. Difficult to enforce ACLs due to annual reporting of catch by fishermen - don't know how much was caught until 6 months or a year later - difficult to monitor, no current way to collect recreational fishery data.

13) Bycatch is monitored

Score: 0 – No mention of bycatch observations. Difficult to enforce size limits, gear restrictions, etc.

14) Bycatch is minimized

Score: 0.5 – actions to reduce bycatch (e.g. gear restrictions, area closures, timing restrictions) are considered. Often harvest everything that is caught.

15) Sensitive habitats are identified and mapped

Score: 1 – Sensitive habitats are identified and mapped. Increased funding from Coral Reef Conservation Program to map coral reef habitat

16) Sensitive habitats are protected

Score: 1 – Sensitive habitats are protected from all use. Funding from Coral Reef Conservation Program mapping used to monitor closed areas.

17) Ecosystem models are available

Score: 1 – Ecosystem models are available for tactical use (explore policies). Ecopath with Ecosim models available in Puerto Rico and the greater Caribbean region.

18) Ecosystem models are used in evaluating policies

Score: 0 – No ecosystem models are used to evaluate policies.

10. Kenyan Pelagic Fishery

Maina, G.W. 2012. A Baseline Report for the Kenyan Small and Medium Marine Pelagic Fishery. Available at: http://www.swiofp.net/publications/component-reports/component-6/fisheries-management/a-baseline-report-for-the-kenyan-small-and-medium-marine-pelagic-fishery.

Ministry of Fisheries Development. A management plan for fisheries targeting small and medium sized pelagic fish. March 2013. http://extwprlegs1.fao.org/docs/pdf/ken147842.pdf

1) Management plan defines the bounds of the ecosystem

Score: 1 – The South Western Indian Ocean Fisheries Project (SWIOFP) is one of three wings of the Agulhas and Somali Currents Large Marine Ecosystems (ASCLME) Programme. One of the main goals of SWIOFP is to clearly define the ecosystem boundaries to address shared, transboundary, migratory fish stocks of the Western Indian Ocean. The three modules of the ASCLME programme collects data on the ocean-atmosphere interface, fisheries, coastal populations, and critical habitats in the region.

2) Ecosystem-based goals

Score: 0.5 – Non-specific ecosystem goals. The government of Kenya states that the objectives of the pelagic management plan are to ensure long-term biological sustainability, ensure the fishery development addresses community, national, and regional concerns and interests, optimize sustainable fishery utilization and benefits, and improve governance. These are not ecosystem-specified, but meeting these general goals would help to meet ecosystem-specific goals that are not explicitly listed.

3) Goals emerge from participatory process

Score: 1 – Stakeholders involved in decision making. In 2007, Kenya mandated co-management through the Fisheries Beach Management Units (BMU) regulations. BMUs were set up to promote cooperation among fishermen and their participation in fisheries management and landings areas. Fishermen have co-management rights. Article 69 of the Constitution of Kenya Act (2010) states that the country seeks to ensure sustainable exploitation, utilization, management, and conservation of natural resources, and in meeting that goal, encourage public participation in the management, protection, and conservation of the environment.

The management plan for the small and medium pelagics fishery was later developed, involving stakeholders to make sure they understood the objectives of the fishery management and the process for decision-making. This is all under the jurisdiction of the Fisheries Bill in 2012 that recognized management plans should be developed with the participation of stakeholders to ensure ownership and compliance. Stakeholders are also involved in stakeholder workshops to discuss policy objectives. Revision of co-management approaches (BMUs) also involve stakeholders. Key stakeholder workshops are also used to address issues with the open access system. The management plan also states that the Kenyan Fisheries Department will "regulate, promote, support, and guide the implementation of the plan, through broad consultative processes with other stakeholders".

4) Considers the impact on humans (economic, cultural, social)

Score: 1 – Uses social-ecological-systems or other social-ecological-economic system. The management plan discusses how improved fisheries management will help reduce the proportion of the human population suffering hunger and malnutrition. The plan states that "the overall purpose of the plan is to ensure that the artisanal fishery targeting small and medium sized pelagic fish in Kenya is sustainable in

the long term, providing the maximum social and economic benefit to the fishers that depend on fishing for their livelihood". The plan is linked to the government's Kenya Vision 2030 social-economic and political development mission.

5) Process for evaluation and adaptability of the management plan

Score: 0.5 – Local level legislative adaptability and evaluation. The National Task Group set up a detailed strategy to ensure management objectives are met, but this was at a national level. Because this was not an international plan for a transboundary, pelagic fishery, I categorized the process for evaluation at the local level only. There is also a relatively lengthy process to change the management plan, which shows some opening for adaptability, but it is relatively inflexible.

6) Management plan recognizes uncertainty and makes allowances

Score: 0 – Does not acknowledge uncertainty. No discussion of uncertainty in the management plan.

7) Interaction of multiple species are considered

Score: 0 – Single species. While some details are available for different species in the "small pelagics" and "medium pelagics" groups, there are no details on how these species interact even if they are harvested by the same gears.

8) Tradeoffs in ecosystem services are evaluated

Score: 0.5 – Ecosystem services are identified but not measured. The impact of where the gears are used (nearshore vs. offshore) is important in this fishery, and the ring net management plan attempts to address this issue. However, the impact of gears in different areas does not appear to be directly measured.

9) Specific ecosystem targets

Score: 0 – No mention of ecosystem targets.

10) Fisheries-independent data collection and monitoring of more than target species

Score: 1 – Independent data collection available for target and non-target species. Biological, social, and economic data collection on top of fishery-dependent data collection. There is some monitoring, control, and surveillance efforts, however difficulty getting all fishers to register with their gear and capacity.

11) Harvest control rules including non-target species

Score: 0 –No specification of harvest control rules, and definitely not for non-target species.

12) Evidence that regulations are effectively enforced

Score: 0.5 – Mentions how regulations are enforced (e.g. listed resources such as boats and workforce). Regulations are supposed to be enforced via registration of fishers with their gear and capacity through trained officers and equipment. However, the Kenyan government suspects low registration rates that require urgent attention.

13) Bycatch is monitored

Score: 0.5 – Bycatch is acknowledged, but not well-quantified. There is knowledge of bycatch with the newly introduced ring net gear when improperly used in nearshore areas. However, some bycatch is known as a benefit as people do not discard it but instead bring it home for subsistence.

14) Bycatch is minimized

Score: 0.5 – Actions to reduce bycatch (e.g. gear restrictions, area closures, timing restrictions) are considered. While the introduction of the new ring net gear was meant to minimize bycatch by using the ring nets offshore, there is more bycatch using ring nets when improperly used inshore.

15) Sensitive habitats are identified and mapped

Score: 1 – Sensitive habitats are identified and mapped. Nearshore coral reef areas are well mapped.

16) Sensitive habitats are protected

Score: 0.5 – Sensitive habitats are protected but some use is still allowed. Nearshore coral reef areas have some protection via community-based marine protected areas. In these cases, the BMUs get to decide where people can fish and which areas will remain closed to fishing.

17) Ecosystem models are available

Score: 0 – No ecosystem models available.

18) Ecosystem models are used in evaluating policies

Score: 0 – No ecosystem models available.

11. Industrial Atlantic tuna fisheries

ICCAT. 2014. 2015-2020 SCRS Science Strategic Plan. Appendix 10. ICCAT Report 2014-2015 (I). Available at: https://www.iccat.int/Documents/SCRS/STRATEGIC-PLAN_EN.pdf.

1) Management plan defines the bounds of the ecosystem

Score: 1 – Full spatial and partial trophic considerations. The boundaries are defined as the epipelagic ecosystem. Some of components of the ecosystem are mentioned in the Science Strategic Plan such us commercial and non-commercial species, environmental effects, fishing impacts on the ecosystem, socio economic aspects, among others.

2) Ecosystem-based goals

Score: 0.5 –The goal 3 in the Science Strategic plan is to give ecosystem based fisheries advice for management of tuna and tuna related species. This goal is very broad and there are no specifications of how implement it or enforce it or what EBFM implies.

3) Goals emerge from participatory process

Score: 0.5 – The goals emerge from the participation of the country members of the Commission, but the participation of other actors, such us NGOs or fishing industry, is limited.

4) Considers the impact on humans (economic, cultural, social)

Score: 0 – The socioeconomic aspects are mentioned in the Science Strategic plan but not yet considered for management.

5) Process for evaluation and adaptability of the management plan

Score: 1 – Regular meetings are performed by the Commission in order to evaluate the performance of different management procedures. Regulations are constantly adapted in order to provide a better management advice based in new data and model outputs.

6) Management plan recognizes uncertainty and makes allowances.

Score: 0.5 – Improvement of data collections are considered in order to reduce uncertainties in the assessments. The first goal in the Science Strategy plan is to quantify the major uncertainties affecting stock assessment and management advice. But, the plan also mentioned that different sources of uncertainty still need to be identified, integrated and evaluated (e.g. stock assessment process uncertainty, biological and environmental uncertainty, management uncertainty).

7) Interaction of multiple species are considered

Score: 0 – Multiple species interactions are not considered for management advice. Management is based in single species assessments.

8) Tradeoffs in ecosystem services are evaluated

Score: 0 – There is no mention of ecosystem services in the Science Strategy plan.

9) Specific ecosystem targets

Score: 0.5 – Some ecosystem targets are stated in the management plan but limited to bycatch mitigation measures. Some mitigations measures have been implemented in some fisheries but their performance or ecosystem impacts have not been evaluated.

10) Fisheries-independent data collection and monitoring of more than target species

Score: 0 – Outside a few independent surveys for the target Bluefin tuna, there is no independent data collection used for management advice.

11) Harvest control rules including non-target species.

Score: 0 – There are no harvest control rules for target or non-target species.

12) Evidence that regulations are effectively enforced.

Score: 0.5 – For target species evidence exists that the implementation of quotas is driving stocks to recover. For by-catch species the evidence is not as clear.

13) Bycatch is monitored

Score: 1 – Bycatch is monitored by different on-board observed programs implemented by each country member of the Commission.

14) Bycatch is minimized

Score: 0.5 – Actions to reduce bycatch (e.g. gear restrictions, area closures) have been recommended and applied in different fleets but not in all of them. For example, tori-lines are required on all longline vessels targeting tunas to reduce seabirds bycatch. However, circle hooks are not required in all fleets to reduce sea turtles by-catch.

15) Sensitive habitats are identified and mapped

Score: 1 – Potential sensitive habitats for by-catch are identified and mapped. For example, areas of high concentration of juveniles of different tuna species and areas of by-catch for sea turtles and sea birds have been identified.

16) Sensitive habitats are protected

Score: 0.5 – A very small fraction of the habitat is temporarily protected by seasonal closures (e.g. to protect juveniles of different species of tunas).

17) Ecosystem models are available

Score: 0 – No ecosystem models are available at the moment.

18) Ecosystem models are used in evaluating policies

Score: 0 – No ecosystem model exists.

12. Southern Bluefin tuna fishery

CCSBT. 2015. Strategic Plan for the Commission for the Conservation of Southern Bluefin Tuna. 2015-2020. Available at: https://www.ccsbt.org/sites/ccsbt.org/files/userfiles/file/docs_english/operational_resolutions/CCSBT_Strategic_Plan.pdf.

1) Management plan defines the bounds of the ecosystem?

Score: 1 – Full spatial and partial trophic considerations. The boundaries of the ecosystems are defined by the boundaries of the jurisdiction of the Commission for the Conservation of Southern Bluefin Tuna (CCSBT). Some of components of the ecosystem are mentioned in the Strategic Plan, in particular by-catch species, environmental components and impact of fishing activities in the ecosystem.

2) Ecosystem-based goals?

Score: 0.5 – Ecosystem based fisheries management is one of the medium priority goals in the CCSBT strategic plan.

3) Goals emerge from participatory process?

Score: 0.5 – The goals emerge from country members of the Commission, but other actors are not deeply involved in the definition of management goals.

4) Considers the impact on humans (economic, cultural, social)?

Score: 0 – Social and economic impacts are not considered and they are not part of the strategic plan.

5) Process for evaluation and adaptability of the management plan?

Score: 1 – Regular meetings are performed by the Commission in order to evaluate the performance of different management procedures. Regulations are constantly adapted in order to provide better management advice based on new information.

6) Management plan recognizes uncertainty and makes allowances.

Score: 0.5 – Some sources of uncertainty are considered in their management procedure. However, significant uncertainty regarding unaccounted mortalities from countries not members of the Commission, and other sources of uncertainty (e.g. biological and environmental) have been not considered.

7) Interaction of multiple species are considered

Score: 0 – Single species model. Multiple species interactions are not considered for management advice.

8) Tradeoffs in ecosystem services are evaluated

Score: 0 – There is no mention of ecosystem services in the strategic plan.

9) Specific ecosystem targets

Score: 0 – Ecosystem targets are not specified in the strategic plan.

10) Fisheries-independent data collection and monitoring of more than target species

Score: 0.5 – There is some independent data collection available but only for the target species (e.g. aerial surveys, Close-kin mark-recapture).

11) Harvest control rules including non-target species.

Score: 0.5 – There are harvest control rules only for the target species.

12) Evidence that regulations are effectively enforced.

Score: 1 – Yes, Southern Bluefin tuna is showing a decrease in fishing mortality and signs of rebuilding.

13) Bycatch is reported and monitored.

Score: 1 – The observed program monitors by-catch.

14) Bycatch is minimized

Score: 0.5 – Actions to reduce bycatch have been considered but not formally implemented.

15) Sensitive habitats are identified and mapped

Score: 0 – No mention of sensitive habitats in the strategic plan.

16) Sensitive habitats are protected

Score: 0 – No mention of sensitive habitats in the strategic plan.

17) Ecosystem models are available

Score: 0 – No ecosystem models are available at the moment.

18) Ecosystem models are used in evaluating policies

Score: 0 – No ecosystem model exists.

13. Lake Victoria Artisanal Fishery

Bwathondi, P.O.J., R. Ogutu Ohwayo, and J. Ogaari. 2014. Lake Victoria fisheries management plan, Lake Victoria Fisheries Organization (LVFO). Available at: http://aquaticcommons.org/4936/1/Fisheries_Management_Plan_I.pdf.

1) Management plan defines the bounds of the ecosystem

Score: 1 – All fisheries within the lake are included

2) Ecosystem-based goals

Score: 1 – Explicit plan to improve the ecosystem health of the lake by coordinating management activities, enforcement and data collection.

3) Goals emerge from participatory process

Score: 1 – Proposed co-management at the local and national levels, including community participation.

4) Considers the impact on humans (economic, cultural, social)

Score: 1 – Explicitly aimed at improving socio-economic status of people who rely on Lake Victoria fisheries.

5) Process for evaluation and adaptability of the management plan

Score: 0.5 – Management plan is very general, with multiple proposals, some would provide for better adaptability than others.

6) Management plan recognizes uncertainty and makes allowances

Score: 0.5 – Some proposals are more precautionary than others, but since none were adopted, no allowances were made.

7) Interaction of multiple species are considered

Score: 1 – Management is at the lake-wide level, including all commercially valuable species, or species that have ecosystem importance to commercially valuable species.

8) Tradeoffs in ecosystem services are evaluated

Score: 0.5 – The proposals include various trade-offs and how to balance different competing interests.

9) Specific ecosystem targets

Score: 0 – None.

10) Fisheries-independent data collection and monitoring of more than target species

Score: 1 – Current trawl surveys exist, and the multiple jurisdictions involved would require monitoring throughout the lake, most likely by an independent group.

11) Harvest control rules including non-target species

Score: 0 – None.

12) Evidence that regulations are effectively enforced

Score: 0 – Never adopted.

13) Bycatch is monitored

Score: 0.5 – There is some effort to identify bycatch, but no lake-wide system, and large areas where no data exist.

14) Bycatch is minimized

Score: 0 – Gillnets are the primary method of catching fish, and virtually any fish are valuable.

15) Sensitive habitats are identified and mapped

Score: 0 – Nothing has been done to identify sensitive habitats.

16) Sensitive habitats are protected

Score: 0 – No protections exist.

17) Ecosystem models are available

Score: 0 – No ecosystem models available.

18) Ecosystem models are used in evaluating policies

Score: 0 – No ecosystem models available.

14. Central Baltic Herring Fishery

Fiskesekretariatet (FISH) and Coalition Clean Baltic. 2014. Joint CCB and FISH considerations regarding ecosystem-based multi-species management in the Baltic Sea. Available at: http://www.fishsec.org/app/uploads/2016/01/140721-FISH-and-CCB-briefing-on-Baltic-ecosystem-based-multispecies-management.pdf.

ICES. 2013. Herring in Subdivisions 25-29 and 32 (excluding Gulf of Riga) Advice for 2014. Available at: http://www.ices.dk/sites/pub/Publication%20Reports/Advice/2013/2013/her-2532-Ex-Go_201304130022.pdf.

1) Management plan defines the bounds of the ecosystem

Score: 1 – Physical boundaries (i.e. ICES convention area) and some biological links clearly defined (i.e. food competition among cod, sprat and herring).

2) Ecosystem-based goals

Score: 0.5 – No explicit ecosystem goals are defined, but some considerations are described in the development of an ecosystem-based multispecies management plan in the Baltic Sea (i.e. include the main actors and their interactions, main environmental drivers and human pressures affecting the ecosystem and how the interactions have changed over time). Multispecies considerations are also described in the assessment.

3) Goals emerge from participatory process

Score: 0 – Different countries are involved in this fishery, but there are no references about other actors working in the definition of the management goals.

4) Considers the impact on humans (economic, cultural, social)?

Score: 0 – Not for management purposes.

5) Process for evaluation and adaptability of the management plan?

Score: 1 – The stock is regularly assessed and precautionary fishing mortality (F_{pa}) separate from F_{MSY} is implemented.

6) Management plan recognizes uncertainty and makes allowances.

Score: 1 – In addition to the previous paragraph, stochastic simulations are performed to account for uncertainty.

7) Interaction of multiple species are considered

Score: 1 – The interaction herring-sprat-cod is considered.

8) Tradeoffs in ecosystem services are evaluated

Score: 0 – Ecosystem services are not evaluated.

9) Specific ecosystem targets

Score: 0 – Specific ecosystem targets are not clearly specified in the management plan; only multispecies interactions are considered.

10) Fisheries-independent data collection and monitoring of more than target species

Score: 1 – Acoustic and bottom trawl surveys and environmental monitoring.

11) Harvest control rules including non-target species.

Score: 0 – No HCR's outside of herring-sprat-cod.

12) Evidence that regulations are effectively enforced.

Score: 0.5 – No mention of enforcement in the reports, but there is a clear reduction in fishing pressure and increase in biomass in the last decade.

13) Bycatch is monitored

Score: 0 – No mention of it in the reports.

14) Bycatch is minimized

Score: 0 – No mitigation methods are used or none are known to be used.

15) Sensitive habitats are identified and mapped

Score: 1 – In the Baltic sea sensitive habitats are well identified and mapped. There are designated sites with particular nature values as protected areas.

16) Sensitive habitats are protected

Score: 1 – MPAs are defined and human activities within those areas are managed. Each site has its unique management plan. http://www.helcom.fi/action-areas/marine-protected-areas

17) Ecosystem models are available

Score: 0 – No ecosystem models are available.

18) Ecosystem models are used in evaluating policies

Score: 0 – A multispecies approach is considered, but no ecosystem models are used.

15. Pacific Halibut fishery

IPHC. 2014. International Pacific Halibut Commission Annual Report 2013. Int. Pac. Halibut Comm. Report of Assessment and Research Activities 2014: 96 p. Available at: http://www.iphc.int/publications/annual/ar2013.pdf

IPHC. 2015. Report of Assessment and Research Activities 2014. Int. Pac. Halibut Comm. Report of Assessment and Research Activities 2014: 653 p. Available at: http://www.iphc.int/publications/rara/2014/rara2014_150109.pdf

1) Management plan defines the bounds of the ecosystem?

Score: 0.5 – The physical fishery limits stretches from AK to CA. However, no trophic or other ecosystem bounds are mentioned in the management plan.

2) Ecosystem-based goals?

Score: 0 – No specific ecosystem goals are defined for this fishery in the management plan.

3) Goals emerge from participatory process?

Score: 0 – There are no participatory processes involved in the definition of the management goals.

4) Considers the impact on humans (economic, cultural, social)?

Score: 0.5 – Part of the managements goals include socio-economic aspects such us fishery sustainability and stability, assurance of access and serve consumer needs.

5) Process for evaluation and adaptability of the management plan?

Score: 1 – Regular assessment are performed in order to evaluate different management procedures. Regulations are constantly adapted in order to provide a better management advice based in new data and model outputs.

6) Management plan recognizes uncertainty and makes allowances.

Score: 1 – Assessment staff works at determining and reducing the level of uncertainty associated with stock assessments through advanced analytical techniques. Where needed, improved data collection or other studies are recommended. Some uncertainties identified are: fishing configuration changes, fleet movements, estimations of discards, etc.

7) Interaction of multiple species are considered

Score: 0 – There is no mention of it in the management plan. The only species considered is Halibut.

8) Tradeoffs in ecosystem services are evaluated

Score: 0 – No mention of it.

9) Specific ecosystem targets

Score: 0 – Ecosystem targets are not specified in the management plan.

10) Fisheries-independent data collection and monitoring of more than target species

Score: 1 – Collection of juvenile abundance data from trawl surveys, which is incorporated into the annual assessment. Fishery dependent data is also collected by a commercial fishery port sampling program.

11) Harvest control rules including non-target species.

Score: 0 – Harvest control rules only exist for the target species.

12) Evidence that regulations are effectively enforced.

Score: 1 – The evidence is reflected in the status of the Pacific halibut population. It is currently not overfished.

13) Bycatch is monitored

Score: 1 – Halibut bycatch is monitored in different fisheries. Also, in the Halibut fishery, the on-board observed programs monitors bycatch of different species.

14) Bycatch is minimized

Score: 0.5 – Alternative hook types to reduce bycatch, such as circle hooks, have been proposed for this fishery. Their effectiveness is still being evaluated.

15) Sensitive habitats are identified and mapped

Score: 0.5 – Some nursery habitats have been identified for Pacific Halibut.

16) Sensitive habitats are protected

Score: 0.5 – Some seasonal and year round area closures for groundfish fisheries have reduced halibut bycatch.

17) Ecosystem models are available

Score: 0 – No ecosystem models exist for this area.

18) Ecosystem models are used in evaluating policies

Score: 0 – No models exist.

16. Baja Yellowtail Fishery

Joubert, E. 2014. Monterey Bay Aquarium Seafood Watch. California Yellowtail, Seriola lalandi (Seriola dorsalis), Isla Natividad, Mexico, Caught by Sociedad Cooperativa de Producción Pesquera Buzos y Pescadores de Baja California, S.C.L. Available at: http://www.seafoodwatch.org/-/m/sfw/pdf/reports/mba_seafoodwatch_mexicocayellowtailreport.pdf.

1) Management plan defines the bounds of the ecosystem

Score: 0.5 – Managed as a part of a larger group of fish at the Federal level, no ecosystem management at local level because finfish are not a primary target for the local fisheries

2) Ecosystem-based goals

Score: 0 – There are not specific ecosystems goals in this fishery

3) Goals emerge from participatory process

Score: 1 – Managed at the local level via fisher owned cooperative in conjunction with top-down federal regulation.

4) Considers the impact on humans (economic, cultural, social)

Score: 0.5 – Impacts are considered via cooperative process, but no formal consideration is made.

5) Process for evaluation and adaptability of the management plan

Score: 0.5 – There is not a formal management plan, but annual take is regulated, and marine protected areas are moved and enforced as needed.

6) Management plan recognizes uncertainty and makes allowances

Score: 0 – There is not a formal plan as the fishery is secondary, and catches are relatively low.

7) Interaction of multiple species are considered

Score: 0.5 – Managed as a complex of similar fish, but since there is no formal management plan, interactions are not really considered. Finfish in this area are caught when it is convenient or when other fisheries are closed.

8) Tradeoffs in ecosystem services are evaluated

Score: 0 – No ecosystem services are evaluated.

9) Specific ecosystem targets

Score: 0 –There are no ecosystem targets.

10) Fisheries-independent data collection and monitoring of more than target species.

Score: 0 – Fisheries collect data on yellowtail, and other species, but little independent data exists, and no stock assessment exists for yellowtail.

11) Harvest control rules including non-target species

Score: 0 – Fishery is of secondary importance to benthic invertebrate fisheries and therefore no rules on finfish harvest are in place beyond permitting.

12) Evidence that regulations are effectively enforced

Score: 1 – The cooperative is very good at enforcing permitting in the area.

13) Bycatch is monitored

Score: 0 – By-catch is retained but no data exists on bycatch rates or species.

14) Bycatch is minimized

Score: 1 – Gillnets are banned and fishing is primarily by hand-line, which effectively minimizes bycatch. This is de facto minimization as there are no apparent rules on type of gear used. Hand lining is likely the cheapest option and is also less likely to impact other local fisheries considered more important.

15) Sensitive habitats are identified and mapped

Score: 1 – Sensitive areas are mapped by the cooperative with the advice of scientists.

16) Sensitive habitats are protected

Score: 0.5 – Areas are protected, but primarily for benthic invertebrates. There may be some benefit to yellowtail, but that is incidental to the real purpose of the protected areas.

17) Ecosystem models are available

Score: 0 – No ecosystem models are available at the moment.

18) Ecosystem models are used in evaluating policies

Score: 0 – No ecosystem model exists.

17. North Sea Cod Fishery

Jardim, E., and F. Scott [ED.]. 2016. Scientific, Technical and Economic Committee for Fisheries (STECF) – Multiannual plan for demersal fisheries in the Western Mediterranean (STECF-16-21); Publications Office of the European Union, Luxembourg; EUR 27758 EN; doi:10.2788/103428. Available at: https://stecf.jrc.ec.europa.eu/documents/43805/1491450/STECF+16-21+-+MAP+demersals+Western+MED.pdf.

ICES, 2014. Cod in Subarea IV (North Sea) and Divisions VIId (Eastern Channel) and IIIa West (Skagerrak) Advice for 2015. Available at: http://www.ices.dk/sites/pub/Publication%20Reports/Advice/2014/2014/cod-347d.pdf

1) Management plan defines the bounds of the ecosystem?

Score: 1 – Physical boundaries (i.e. ICES convention area) and biological links are considered (i.e. mix fisheries and multispecies interactions).

2) Ecosystem-based goals?

Score: 0 – No explicit ecosystem goals are defined. The only reference related to this points says: "The multiannual plans should establish the framework for the sustainable exploitation of stocks and marine ecosystems concerned…".

3) Goals emerge from participatory process?

Score: 0 – Different countries are involved in this fishery, but there are no references about other actors working in the definition of the management goals.

4) Considers the impact on humans (economic, cultural, social)?

Score: 0 – No for management purposes.

5) Process for evaluation and adaptability of the management plan?

Score: 1 – The stock is regularly assessed and precautionary fishing mortality (Fpa) separate from FMSY is implemented.

6) Management plan recognizes uncertainty and makes allowances.

Score: 1 – Yes, simulations studies are evaluated.

7) Interaction of multiple species are considered

Score: 0.5 – Only the interaction herring-sprat-cod is considered for management.

8) Tradeoffs in ecosystem services are evaluated

Score: 0 – Ecosystem services are not evaluated.

9) Specific ecosystem targets

Score: 0 – Specific ecosystem targets are not clearly specified in the management plan. Only multispecies interactions are considered.

10) Fisheries-independent data collection and monitoring of more than target species

Score: 1 – Acoustic and bottom trawl surveys.

11) Harvest control rules including non-target species.

Score: 0 – No HCR's for non-target species.

12) Evidence that regulations are effectively enforced.

Score: 0.5 – No mention of enforcement in the reports, but there is a clear reduction in fishing pressure and increase in biomass in the last decade for cod.

13) Bycatch is monitored

Score: 0 –The two major bycatch species, redeye round herring and horse mackerel, are subject to annual quotas. However, there is no mention of monitoring of bycatch it in the reports.

14) Bycatch is minimized

Score: 0.5 – The gillnet fishery for cod takes bycatches of harbour porpoise. Since 2001, effort reductions in this fishery have likely led to a decrease in bycatches. Also, the two major bycatch species, redeye round herring and horse mackerel, are subject to annual quotas.

15) Sensitive habitats are identified and mapped

Score: 0 – No mention of it in the reports.

16) Sensitive habitats are protected

Score: 0 – No mention of it in the reports

17) Ecosystem models are available

Score: 0 – No ecosystem models are available.

18) Ecosystem models are used in evaluating policies

Score: 0 – A multispecies approach is considered, but no ecosystem models are used.

18. Great Lakes Whitefish

Bunnell, D.B. [ED.]. 2012. The state of Lake Michigan in 2011. Great Lakes Fish. Comm. Spec. Pub. 12-01. Available at: http://www.glfc.org/pubs/SpecialPubs/Sp12_1.pdf

Casselman, J.M., K.T. Scribner, and G.R. Spangler. 2001. Summary of Presentation Review of lake whitefish stocks in northern Lake Michigan, with special reference to stock structure and spawning site distribution in relation to Green Bay. Available at: http://www.glfc.org/lakecom/lmc/lmbg_ws.pdf.

1) Management plan defines the bounds of the ecosystem

Score: 0 – No definition of bounds. Moreover, management plans are at the state level, no co-management plans have been implemented, though the Great Lakes Fishery Commission does exist to potentially coordinate a plan.

2) Ecosystem-based goals

Score: 0 – No specific ecosystems based goals.

3) Goals emerge from participatory process

Score: 0 – Fishery planning is from state and provincial agencies and Tribes. There is a regional council, but it has an advisory role only. There is no formal mechanism by which communities can have input on fishing.

4) Considers the impact on humans (economic, cultural, social).

Score: 0.5 – Only economic factors are considered in management decisions.

5) Process for evaluation and adaptability of the management plan

Score: 0.5 – Since there is no overarching management plan, there is nothing to evaluate, but there is adaptation based on stock assessments at the state level.

6) Management plan recognizes uncertainty and makes allowances

Score: 0.5 – The fisheries seem to use conservative catch allowances because overall stock size is unclear.

7) Interaction of multiple species are considered

Score: 0 – No consideration of other species.

8) Tradeoffs in ecosystem services are evaluated

Score: 0 – None.

9) Specific ecosystem targets

Score: 0 – None.

10) Fisheries-independent data collection and monitoring of more than target species

Score: 0 – None.

11) Harvest control rules including non-target species.

Score: 1 – TACs and quotas exist for state and tribal fisheries. Bycatch is unknown, though other commercial species are managed in similar ways.

12) Evidence that regulations are effectively enforced.

Score: 1 – Despite the number of jurisdictions involved, regulations appear to be enforced fairly well

13) Bycatch is monitored.

Score: 1 – Bycatch seems to be minimal, and largely consists of other commercially valuable species.

14) Bycatch is minimized

Score: 1 – Due to the deep-water schooling behavior of whitefish, bycatch should be minimized.

15) Sensitive habitats are identified and mapped

Score: 0 – Habitats have not been identified.

16) Sensitive habitats are protected

Score: 0 – Sensitive habitats are not protected.

17) Ecosystem models are available

Score: 0 – None exist.

18) Ecosystem models are used in evaluating policies

Score: 0 – Not used.

19. Ecuadorian Artisanal Fisheries

Arriaga, L., and J. Martinez. 2002. Subsecretaria de Recursos pesqueros. Plan de ordenamiento de la pesca y acuicultura del Ecuador. 116 p. Available at: http://oa.upm.es/14340/2/Documentacion/1_Memoria/PlanOrdenacionPescaEcuador.pdf.

1) Management plan defines the bounds of the ecosystem

Score: 0.5 – The ecosystem is defined as the continental coastline. Some ecosystems such as mangroves, lagoons and coastal wetlands are also identified. However, there is no mention of bounds related to trophic levels or trophic interactions.

2) Ecosystem-based goals?

Score: 0 – No mention of specific ecosystem goals in the management plan.

3) Goals emerge from participatory process

Score: 1 – The management plan emphasized an open participation process to public and private sectors in fisheries management (e.g. shrimp fisheries and recreational fisheries promote the involvement of local fishermen in the establishment of regulatory and management measures).

4) Considers the impact on humans (economic, cultural, social)

Score: 0.5 – In the management plan, one of the projects promotes the organization of fishermen, either by communities or by type of fishery, respecting their autonomy and other local cultural or economic characteristics.

5) Process for evaluation and adaptability of the management plan

Score: 0 – No built-in adaptability or evaluation mentioned in the management plan.

6) Management plan recognizes uncertainty and makes allowances.

Score: 0 – No mention of sources of uncertainty related with fisheries management.

7) Interaction of multiple species are considered

Score: 0 – Multiple species interactions are not considered for management advice.

8) Tradeoffs in ecosystem services are evaluated

Score: 0.5 – Ecosystem services are identified (e.g. food production and cultural benefits), but tradeoffs are not evaluated.

9) Specific ecosystem targets

Score: 0 – Ecosystem targets are not specified in the strategic plan.

10) Fisheries-independent data collection and monitoring of more than target species

Score: 0 – There is some independent data collection available but only for target species. However, this information is not available and not mentioned in the management plan.

11) Harvest control rules including non-target species.

Score: 0 – There is no mention of any harvest control rules in the management plan.

12) Evidence that regulations are effectively enforced.

Score: 0 – No public evidence that the regulations in place are enforced.

13) Bycatch is reported and monitored.

Score: 0.5 – Some observed programs, mainly implemented by different NGOs, monitors by-catch in artisanal fisheries. However, the percentage of coverage is probably very low.

14) Bycatch is minimized

Score: 0.5 – Actions to reduce bycatch in some fisheries have been implemented. For example, circle hooks are used in artisanal fisheries to reduce sea turtles bycatch.

15) Sensitive habitats are identified and mapped

Score: 1 – Sensitive habitats like mangroves, lagoons, corals, coastal wetlands and areas of reproduction of different aquatic species are identified and mapped.

16) Sensitive habitats are protected

Score: 0.5 – Some of the habitats mentioned above are protected by marine protected areas and reserves.

17) Ecosystem models are available

Score: 0 – No ecosystem models are mentioned in the management plan.

18) Ecosystem models are used in evaluating policies

Score: 0 – No ecosystem models are available at the moment.

20. Washington spot shrimp Fishery

Wargo, L., D. Ayres, and Y.W. Cheng. 2013. Washington Coastal Spot Shrimp Fishery. Washington Department of Fish and Wildlife Fish Program Fish Management Division. Fish Program Report Number FPT 13-01. Available at: http://wdfw.wa.gov/publications/01532/wdfw01532.pdf

1) Management plan defines the bounds of the ecosystem?

Score: 0.5 – No physical or trophic ecosystem bounds in the management plan are mentioned. However, shrimp habitat, which ranges from 20 to 40 miles offshore, and fishing areas (Puget Sound and Hood Canal) are defined.

2) Ecosystem-based goals?

Score: 0.5 – No specific ecosystem goals are defined for this fishery in the management plan. However, "ecosystem considerations" is mentioned as one management goal and also "minimize bycatch of other species" and "minimize impacts to habitat".

3) Goals emerge from participatory process?

Score: 0.5 –Some commercial and recreational sectors are involved in the management process, including a few coastal tribes.

4) Considers the impact on humans (economic, cultural, social)?

Score: 0.5 – Some socio-economic considerations are mentioned in the management plan: Tribal co-management, licensing programs and other economic evaluations.

5) Process for evaluation and adaptability of the management plan?

Score: 1 – It has been adapted since 1999 with implementation of TACs, reduction in quotas, seasonal closures and a Limited Entry License Program.

6) Management plan recognizes uncertainty and makes allowances.

Score: 0 – There is no mention of it in the management plan.

7) Interaction of multiple species are considered

Score: 0 – There is no mention of it in the management plan.

8) Tradeoffs in ecosystem services are evaluated

Score: 0 – Ecosystem services are not evaluated in this fishery.

9) Specific ecosystem targets

Score: 0 – Ecosystem targets are not specified in the management plan.

10) Fisheries-independent data collection and monitoring of more than target species

Score: 0 – There are no fisheries independent data collection programs.

11) Harvest control rules including non-target species.

Score: 0.5 – They have control rules only for target species.

12) Evidence that regulations are effectively enforced.

Score: 0.5 – TACs are effectively enforced for target species but there is no assessment for non-target species.

13) Bycatch is monitored

Score: 0 – Overall, bycatch in the coastal spot shrimp fishery by pot gear is much less than that observed in trawl nets. It's around 30%, mainly invertebrates: urchins, snails and sea stars. However, it is not currently monitored.

14) Bycatch is minimized

Score: 0.5. To reduce bycatch, since 2003, pots are the only legal gear in the fishery. Trawl fisheries for shrimp were banned.

15) Sensitive habitats are identified and mapped

Score: 0 – No mention in the management plan.

16) Sensitive habitats are protected

Score: 0 – See previous paragraph.

17) Ecosystem models are available

Score: 0 – No ecosystem models exist for this area.

18) Ecosystem models are used in evaluating policies

Score: 0 – No models exist.

21. Indonesian Blue Swimming Crab

MRAG Americas, Inc. 2009. Pre-Assessment of the Indonesian Blue Swimming Crab Fishery. Available at: http://www.committedtocrab.org/wp-content/uploads/2015/04/MRAG-Indonesia-Blue-Swimming-Crab-Pre-assessment-2-July-2009.pdf.

1) Management plan defines the bounds of the ecosystem

Score: 0.5 – Bounds set poorly, not reflective of ecosystem. The boundary of the fishery is defined as the EEZ, but the bounds of the ecosystem are not well defined.

2) Ecosystem-based goals

Score: 0.5 – Non-specific ecosystem goals. Among the long-term goals of Indonesia's fishing policy are to manage fisheries resources in a sustainable manner, increasing fisher's income and welfare. While these may be ecosystem-oriented, they are not ecosystem-specific.

3) Goals emerge from participatory process

Score: 0.5 – stakeholders involved but not directly in decision-making. The report says that "management at the municipal level likely involves consultation with local stakeholders, but it is not clear that national or regional management seeks, accommodates, or uses stakeholder input".

4) Considers the impact on humans (economic, cultural, social)

Score: 0 – Social and economic impact not considered. Because there is no specific management plan for blue swimming crab, the management framework does not directly consider the economic, cultural, or social impact.

5) Process for evaluation and adaptability of the management plan

Score: 0 – no built in adaptability or evaluation. The report says that "It seems unlikely that the local governments have the capacity to evaluate fisheries and establish adequate controls for heavily fished stocks, or to coordinate management across jurisdictions, although national involvement could assist in this". While there has been a data-limited assessment on this stock since this report was written, it is unlikely the overall management plan will change very easily. It would likely be a grassroots effort, and there is no indication this is happening.

6) Management plan recognizes uncertainty and makes allowances

Score: 0 – Does not acknowledge uncertainty. Because there is no official management plan, there is no acknowledgment of uncertainty.

7) Interaction of multiple species are considered

Score: 0 – Single-species. While bycatch is discussed, there is no direct discussion of interaction between species.

8) Tradeoffs in ecosystem services are evaluated

Score: 0 – No mention of ecosystem services. There are no specific studies of the impacts of gears or other parts of the fishery on ecosystem services.

9) Specific ecosystem targets

Score: 0 – No mention of ecosystem targets. While there are some estimates of stock density and more recently, a data-limited stock assessment, there are no apparent ecosystem-based targets.

10) Fisheries-independent data collection and monitoring of more than target species

Score: 0 – No independent data collection available. The report notes that fishery-independent stock assessment and sampling is required to determine limit and target reference points, but no fishery-independent data collection currently exists.

11) Harvest control rules including non-target species

Score: 0 – No harvest control rules for non-target species. There are no explicit harvest control rules for the blue swimming crab fishery, much less non-target species also impacted by the fishery.

12) Evidence that regulations are effectively enforced

Score: 0.5 – Mentions how regulations are enforced (e.g. listed resources such as boats and workforce). The report mentions that the enforcement of management agencies is weak, but local communities of coastal fishers have prevented trawlers or written local agreements.

13) Bycatch is monitored

Score: 0.5 – Bycatch is acknowledged, but not well-quantified. There is known bycatch due to the types of gears used to capture blue swimming crab – bottom gillnet and collapsible trap. Each are known to have high levels of retained species and bycatch. However, it is difficult to monitor.

14) Bycatch is minimized

Score: 0 – No mention of effort to minimize or reduce bycatch. While it is known approximately how much of the catch from the blue swimming crab fishery is bycatch, there is no discussion on an effort to minimize the bycatch.

15) Sensitive habitats are identified and mapped

Score: 0.5 – Only some potential habitats are identified and mapped. The report states that there are sensitive habitats (e.g. coral reefs) and that certain gears from the fishery are likely destructive of those habitats.

16) Sensitive habitats are protected

Score: 0 – Sensitive habitats are not protected. The report calls for protection of sensitive habitats meaning that they were not protected when this document was produced.

17) Ecosystem models are available

Score: 0 – No ecosystem models available.

18) Ecosystem models are used in evaluating policies

Score: 0 – No ecosystem models available.
